# Gas-phase synthesis and time-resolved composition analysis of CuZn nanoparticles

**DOI:** 10.1039/d6na00440g

**Published:** 2026-07-03

**Authors:** Linnéa Jönsson, Vinzent Olszok, Dániel Megyeri, Thomas Krinke, Calle Preger, Jenny Rissler, Axel C. Eriksson, Zsolt Geretovszky, Knut Deppert, Alfred P. Weber, Attila Kohut, Maria E. Messing

**Affiliations:** a Solid State Physics, Lund University Lund 221 00 Sweden linnea.jonsson27@gmail.com; b NanoLund, Lund University Lund 221 00 Sweden; c Clausthal University of Technology, Institute of Particle Technology Clausthal-Zellerfeld 38678 Germany; d Department of Optics and Quantum Electronics, University of Szeged Szeged 6720 Hungary; e MAX IV Laboratory, Lund University Box 118 Lund 221 00 Sweden; f Ergonomics and Aerosol Technology, Lund University Lund 221 00 Sweden; g Quantum Device Physics Laboratory, Department of Microtechnology and Nanoscience, Chalmers University of Technology Gothenburg 41296 Sweden

## Abstract

Bimetallic CuZn (brass) nanoparticles are key materials in catalytic applications, yet access to the full compositional range remains challenging using conventional wet chemical synthesis. In this study, we demonstrate the physical synthesis of CuZn nanoparticles across a broad compositional range using spark ablation of alloyed feedstocks (Cu_25_Zn_75_, Cu_50_Zn_50_, Cu_75_Zn_25_). In spark ablation, the nanoparticles are formed directly in the gas phase without the need for post-synthesis treatments and exhibit complete internal mixing, as confirmed by (scanning) transmission electron microscopy ((S)TEM) and energy-dispersive X-ray spectroscopy (EDS). A pronounced evolution over time in nanoparticle composition was observed during continuous generation. To elucidate the underlying mechanisms, a comprehensive set of advanced, time-resolved characterization techniques was employed, including X-ray fluorescence (XRF) of deposited nanoparticles, optical emission spectroscopy (OES) of the spark plasma, in-flight inductively coupled plasma mass spectrometry (ICP-MS), and in-flight X-ray photoelectron spectroscopy (XPS). These complementary characterization methods reveal a gradual compositional evolution linked to changes at the feedstock surface rather than post-formation processes. The results indicate that preferential Zn evaporation governs the temporal evolution of the nanoparticle composition, followed by the establishment of a dynamic steady state during prolonged sparking. Based on the experimental observations, a qualitative mechanism supported by a simple ablation model is proposed to explain the compositional evolution in CuZn spark ablation. Despite the large differences in thermophysical properties between Cu and Zn, a broad Cu–Zn compositional range can be accessed, with stable nanoparticle compositions achieved upon extended operation. This work provides insight into bimetallic nanoparticle formation *via* spark ablation and how tunable alloy compositions can be achieved *via* gas-phase synthesis, with direct relevance for catalytic and other composition-sensitive applications.

## Abstract 2

Time-resolved in-flight analysis reveals the temporal evolution of CuZn nanoparticle composition during spark ablation.

## Graphical abstract

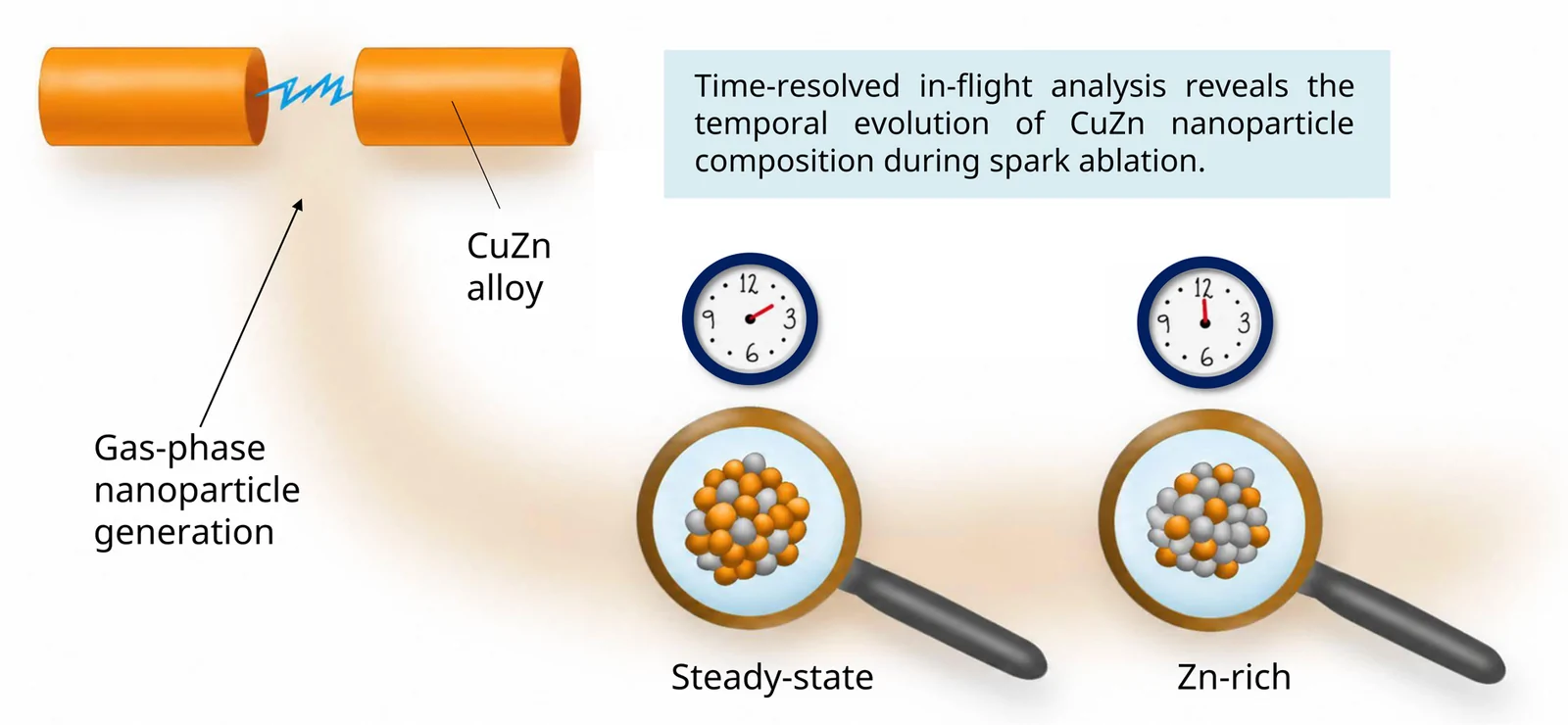

## 1 Introduction

Due to their tunable properties, including size, morphology, and composition, metal and metal oxide nanoparticles (NPs) are widely used in applications such as catalysis, energy storage, medical diagnostics, and sensing. Compared with monometallic NPs, which consist of a single metal/metal oxide, bimetallic NPs offer an additional degree of freedom for tailoring material properties. This added compositional flexibility is particularly advantageous in catalysis and sensing, where changes in elemental composition can significantly affect reactivity, selectivity, and sensitivity.^1,2^ Once an optimal composition has been identified for a given application, it becomes essential to employ a nanoparticle synthesis method capable of precisely controlling composition, ensuring that all particles contribute efficiently to the desired performance.

Bimetallic Cu–Zn (brass) NPs are a prominent example, as they are thought to play an important role in catalyzing the synthesis of methanol from CO_2_-, CO-, and H_2_-containing gas mixtures.^3^ The reaction is particularly interesting because it represents a possibility to produce a sustainable fuel from captured CO_2_ and green H_2_.^4^ The catalytic performance of Cu-based systems in CO_2_ hydrogenation is strongly influenced by structural properties where increased Cu dispersion and smaller crystallite sizes enhance activity.^5^ Consequently, considerable effort has been devoted to developing synthesis routes that improve catalyst dispersion, composition control, and selectivity.

The most common approach for producing bimetallic NPs is wet-chemical synthesis, in which metal precursors are reduced or thermally decomposed in solution in the presence of stabilizing ligands. While such methods enable good size control and are suitable for large-scale production, achieving homogeneous and compositionally tunable CuZn alloys is challenging. Co-reduction or co-decomposition of Cu and Zn precursors typically results in specific intermetallic phases (such as α-, β-, and γ-brass) or mixtures thereof, depending sensitively on precursor ratios, ligands, and reaction conditions, rather than allowing continuous compositional control.^3,6^ Zn-rich compositions are particularly difficult to obtain, as excess Zn readily forms the thermodynamically stable ZnO phase. Similarly, co-precipitation approaches are limited by the different precipitation kinetics of Cu^2+^ and Zn^2+^ ions, often leading to phase-segregated structures and incomplete alloying.^7^

In addition, wet-chemical routes require post-synthesis treatments such as ligand removal, washing, calcination, and reduction. These steps can induce phase separation and promote the formation of distinct CuO, Cu, and ZnO crystallites, thereby reducing metal dispersion and altering the intended alloy structure.^5^ Furthermore, each targeted composition, structure, or particle size typically demands development of new recipes that needs to be optimized. Together, these constraints reflect fundamental methodological, thermodynamic, and kinetic limitations associated with wet-chemical synthesis of CuZn alloy NPs.

Herein, an alternative synthesis route is presented, taking advantage of gas-phase physical synthesis methods which can offer excellent mixing and composition control of the NPs while avoiding liquid-phase processing. A key advantage of gas-phase physical methods, in which NPs are formed from feedstock materials and suspended in a gas, is the inherently ligand-free particle surface, eliminating the need for additional purification steps. Spark ablation stands out compared to other physical nanoparticle generation approaches, such as thermal evaporation, arc discharge, and magnetron sputtering, as it is a particularly versatile method for producing bi- and multimetallic NPs.^8,9^ In spark ablation, short electrical discharges between electrodes ablate atoms from the feedstock materials, which subsequently cool, nucleate, and condense into NPs suspended in the gas phase. NPs can be formed from virtually any electrically conductive material, including combinations of materials that are immiscible at the macroscopic scale.^9,10^ It also possible to tune the particle composition by controlling the feedstock compositions^11^ and generation parameters.^12^

In this study, bimetallic Cu–Zn NPs are generated in the gas phase *via* spark ablation using alloy feedstocks with varying compositions (Cu_25_Zn_75_, Cu_50_Zn_50_, and Cu_75_Zn_25_). The size, morphology and composition of the resulting NPs are characterized by TEM, STEM-EDS, and TEM-EDS. Post-generation analysis of the electrode surfaces after 30 minutes of operation, performed using SEM-EDS, revealed a substantial deviation from the initial feedstock composition, as well as from the nanoparticle compositions. The observations indicate a time-dependent compositional evolution of both electrodes and particles during generation.

Such indications were reported already in an article by Watters *et al.* from 1989. They showed that particles sampled 5 seconds after the start of generation were enriched in Zn relative to the original alloy composition. Correspondingly, elemental analysis of the electrode surface after sparking showed a lower Zn content than before sparking. The effect became more pronounced for particles sampled after 120 seconds, thus indicating a time-dependent evolution of nanoparticle composition during continuous operation.^13^

To elucidate the time-dependent composition behavior, time-resolved characterization techniques were employed. XRF measured continuously deposited NPs, OES probed the temporal evolution of atomic species in the spark plasma, and in-flight ICP-MS provided signals from ^63^Cu and ^64^Zn isotopes in the NPs. The nanoparticle surface composition was investigated using in-flight XPS at the MAX IV synchrotron facility in Lund, Sweden.

The results demonstrate that bimetallic CuZn NPs can be successfully synthesized, and that a wide compositional range of the Cu–Zn system can be accessed using this technique. The compositional tunability is of high importance for tailoring nanoparticle properties, for example in catalytic applications where activity and selectivity are strongly composition dependent. By combining advanced time-resolved characterization techniques, this work provides detailed insight into the compositional evolution of CuZn NPs formed *via* spark ablation, and a qualitative ablation model explaining the time dependent behavior is proposed.

## 2 Experimental section and characterization

The time-resolved measurements were performed using two different spark ablation systems. The two systems are similar but built and placed at different locations (Lund and Clausthal). Both systems have been described in detail elsewhere (Lund:^14^, Clausthal:^15^) and hereafter, these systems are referred to as the Lund and Clausthal setups. Both setups were operated under similar conditions, including a capacitor capacitance (C) of 19 nF (Lund) and 24 nF (Clausthal), an electrode gap of 2 mm, and an Ar carrier gas flow rate of 1 L min^−1^. The CuZn alloy electrodes were custom-made by Goodfellow with specified nominal compositions of Cu_25_Zn_75_, Cu_50_Zn_50_, and Cu_75_Zn_25_, and had a diameter of 3 mm. Certified purity values were not available. The nominal Cu/Zn compositions were verified experimentally by SEM-EDS analysis of polished, unsparked electrode surfaces. Using 
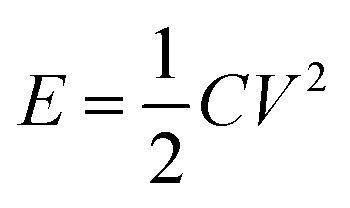
 with a discharge voltage (*V*) of approximately 1.2 kV, the estimated single-discharge energies were 13.7 mJ for the Lund setup and 17.3 mJ for the Clausthal setup. Charging currents of 6 mA and 2 mA were employed, resulting in spark frequencies of ∼130 Hz and ∼43 Hz, respectively, while the spark durations remained below 8 µs for both setups and operating conditions. An exception to these experimental conditions was made when substantially higher particle concentrations were needed to obtain sufficient signal for time-resolved in-flight XPS measurements. For those measurements, nitrogen was used as the carrier gas with a charging current of 30 mA.

### 2.1 Particle generation

To generate the NPs, a pair of electrodes is placed inside a chamber flushed with a carrier gas. The electrodes are connected to a capacitor bank charged using a high-voltage power supply, creating a strong electric field across the gap that eventually causes electrical breakdown of the carrier gas and formation of a conductive plasma. Charge is then rapidly transferred between the electrodes through an oscillatory discharge. During this process, the electrode polarity periodically reverses as the current oscillates, causing negatively charged species (mainly electrons) and positively charged species (mainly ions) to alternately bombard both electrodes. This oscillatory behavior enables elemental mixing when two different electrode materials are used. Since the oscillations are damped over time, the electrode that is initially negative (cathode) typically experiences the highest total ion bombardment and, consequently, the largest material removal. However, the delivered energy can be tuned to some extent by adjusting the circuit characteristics.^12,16,17^

Because ions have a much higher mass and inertia than electrons, material ablation is dominated by ion bombardment. The impact of the charged species on the electrode surface results in sputtering, rapid local heating, melting, and evaporation of material. Most of the delivered energy contributes to melting and redistribution of the electrode material, leading to the formation of erosion craters and resolidified molten structures, while only a small fraction results in material erosion and vaporization that contributes to nanoparticle formation.^18,19^ As the produced vapor cools, the material nucleates into atomic clusters that eventually coalesce and grow into NPs in turn aggregating into larger particles (still in the nano size regime). During formation, the particles are transported away from the spark chamber by the carrier gas.

A significant difference compared to simple thermal evaporation to produce NPs, is that the sparking process involves ablation of atoms into a local hot plasma (∼20 000 K) before they are cooled to room temperature.^20^ The fast quenching (∼10^8^–10^9^ K s^−1^ (ref. 21)) of evaporated material allows for formation of particles made of the same composition as that of the vapor, allowing for atomic mixing on the nanoscale. In other words, difference in condensation temperature between the elements is irrelevant in this process.^8^ Previous work also suggests that distillation effects, *i.e.*, preferential evaporation of one element over another, are suppressed due to the short spark discharge durations, typically below 10 µs, such that ablation results in near-instantaneous material removal.^8,17^ Studies of the Cr–Co and Ag–Au material systems demonstrated particles with compositions close to that of the alloyed electrodes, despite some differences in vapor pressure.^14,22^

### 2.2 Size distribution

The particle size distributions in Ar were qualitatively determined using a differential mobility analyzer (DMA, custom Vienna-type design) coupled to an electrometer (TSI 3068B). Before entering the DMA, the aerosol was passed through a ^63^Ni beta neutralizer to establish an equilibrium bipolar charge distribution. The DMA was operated with an aerosol flow of 1 L min^−1^ and a sheath flow of 10 L min^−1^, and particles were classified according to their electrical mobility before detection by the electrometer. The DMA-electrometer setup was calibrated for N_2_-based carrier gases. No inversion or deconvolution procedure was applied to the measured data; consequently, the DMA transfer function, particle charging efficiency/bipolar charge distribution, and multiply charged particles were not corrected for. The reported distributions should therefore be regarded as apparent mobility distributions and interpreted only qualitatively. They are intended to compare relative differences between particles generated from the three CuZn alloy compositions under identical measurement conditions, rather than to provide absolute size distributions or values directly comparable to fully corrected SMPS measurements.

### 2.3 Single-particle characterization

Investigation of the morphology (TEM), spatial elemental distribution (STEM-EDS), and average composition (TEM-EDS) of individual particles was done using a TEM (JEOL 3000F) equipped with EDS and capable of operation in STEM mode. The NPs were generated using a charging current of 6 mA, and prior to TEM deposition, the aerosol was neutralized and passed through a DMA operated at a fixed voltage corresponding to an electrical mobility diameter of 70 nm. These size-selected particles were deposited 10 min after the start of sparking. Single particles generated using both the Lund and Clausthal setups were investigated. For elemental analysis by EDS, the Inca software package (Oxford Instruments) was used, and the K-lines for Cu and Zn were used for quantification.

For morphology and spatial elemental distribution, particles were generated using the Lund setup and deposited (for ∼3 minutes) on lacey carbon copper (for morphology) or gold (elemental distribution) TEM-grids using a custom electrostatic precipitator, like the one developed by Dixkens and Fissan.^23^ The samples were exposed to ambient atmosphere prior to analysis. Samples generated using the Lund setup for morphology and STEM-EDS were stored for a few hours, whereas samples generated using the Clausthal setup for TEM-EDS composition analysis were stored for a few weeks. Surface oxidation during storage is expected; however, the TEM-EDS analysis is used to determine the average relative Cu/Zn elemental composition, and is therefore not expected to be significantly affected by storage-induced oxidation.

For average composition of individual particles by TEM-EDS, NPs were generated using the Clausthal setup and were diffusively deposited (for ∼5 seconds) on Ni lacey carbon TEM-grids. For each sample, the compositions of 20 particles were measured. Each measurement was done by condensing the electron beam onto a single particle at a magnification of 300 000*X* and collecting a spectrum for 60 live time seconds.

### 2.4 SEM & SEM-EDS of electrodes

The surface structure and composition of the electrodes was analyzed using SEM and SEM-EDS (Shottky FEG type SEM, model GeminiSEM 500 from Zeiss equipped with an Ultim Max 170 detector). The composition of both the sparked (using 6 mA in Ar for 30 minutes) and unsparked (polished with sandpaper) alloyed CuZn electrodes was analyzed using an acceleration voltage of 20 kV. Spectra were acquired for 20 live time seconds, *i.e.*, the effective acquisition time excluding detector dead time, at 30 different positions on the electrode surface both of anodes and cathodes and evaluated in the AZtec software using the K lines of Cu and Zn.

### 2.5 Time-resolved XRF

Time-resolved XRF of the aerosol particles was performed using the Lund setup connected to the “Xact” (Cooper Environmental, Xact 625i); a device that samples an aerosol and use energy dispersive XRF to provide information about the atomic abundance in the sample.^24^ For these measurements, a charging current of 2 mA was used for particle generation. The aerosol consisting of nanoparticles in Ar with a flow of 1 L min^−1^ was diluted with 9 L min^−1^ N_2_ using a mass flow controller. The diluted aerosol was sampled by the Xact onto the instrument's filter roll for 5 or 15 minutes. The sample was then irradiated for 5 or 15 minutes, resulting in a fluorescence spectrum that is evaluated using internal software and provides the average composition of the NPs.

### 2.6 Time-resolved plasma OES

The light emitted from the spark plasma during the particle generation process was coupled into an optical fiber (Thorlabs, M28L02) by using a quartz lens (Avantes COL-UV/VIS) mounted outside the chamber window of the Lund setup. A similar arrangement has been used before to investigate the optical emission of a spark ablation plasma.^21^ The acquired – spatially integrated – emitted light was analyzed by using a compact spectrometer (Avantes, AvaSpec-ULS3648). Optical emission spectra were collected and stored automatically every 10 seconds after starting the sparking with an integration time of 10 ms and averaging of 1000 scans. As a result, the variation of the excited species' intensity could be investigated with a temporal resolution of 10 seconds for a total period of 30 minutes. This approach was used to follow the intensity variation of several spectral lines characteristic to each electrode component and the gas atmosphere (predominantly Cu I 515.32 nm, Zn I 636.23 nm, and Ar I 912.30 nm).

The purpose of these experiments was to detect any variation in the relative emission corresponding to Zn and Cu atoms, potentially indicating variation in the electrode ablation process. Due to the inherent positional instability of the spark plasma and potential variations in light collection efficiency, an atomic argon line was used as internal reference, and Cu I and Zn I intensity variations are therefore presented as ratios with respect to an Ar I line. We assume that Ar, used as carrier gas in the spark chamber, is homogeneously distributed between the electrodes and any potential intensity variation corresponding to Ar I emission is due to the spark's spatial instability and light collection variability.

It should be noted that in addition to the concentration of the excited species, atomic emission is also sensitive to plasma temperature variation.^20^ The present experimental setup does not allow for a meaningful temperature calculation, but the Ar I line can be used to detect indications of considerable plasma temperature variations. To this end, we calculated the intensity ratios of different Ar I lines with different upper energy levels and concluded that the plasma temperature does not vary in a way that could explain the observed intensity variations. Therefore, we concluded that intensity variation of Cu I and Zn I emission can be qualitatively linked to the variation of the relative ablation rate of the corresponding electrode material.

### 2.7 Time-resolved ICP-MS

To qualitatively investigate of how the amount of Cu *versus* Zn in the resulting NPs changes over time, the Clausthal setup was connected to a time-resolved Agilent ICP-MS analyzer (8900 triple quad, Agilent Technologies Inc.), which has been used for analysis of aerosol particles before.^15,25^ To avoid over-loading the plasma, the particle number concentration must be low (∼100 particles per cm^3^). To achieve this, the particles were both size selected at 70 nm using a differential mobility analyzer (DMA, model 3081A, TSI Inc.) and diluted using a rotating disk diluter (RDD, model 379020A, TSI Inc.). In parallel, the number concentration of particles was recorded using a condensation particle counter (CPC model 3750, TSI Inc.), allowing for the signal from the ICP-MS to be corrected for variations in particle number concentration (see Fig. S1). Since the MS detector can only measure one mass signal at a time, signal detection between the Cu and Zn elements was varied 10 times per second, enabling semi-simultaneous measurement of both signal intensities.

### 2.8 In-flight XPS

The Lund setup was transported to the synchrotron facility MAX IV in Lund, Sweden, and connected to the aerosol sample-delivery system at the gas-phase end station of the FinEstBeAMS beamline^26^ for in-flight XPS. Similar in-flight measurements of engineered nanoparticles using the same setup have been conducted before, but without time-resolved analysis.^27–29^ The continuous aerosol flow of CuZn NPs was introduced from near-atmospheric pressure into a vacuum chamber, where the particles are forming a narrow, highly concentrated beam. The aerosols enter the system through a 140 µm critical orifice at a constant mass flow of 0.177 L min^−1^, passed through a relaxation tube, and were subsequently collimated by an aerodynamic lens (Aerodyne PM1). The resulting focused particle beam was transmitted through a 1.5 mm conical skimmer into the vacuum chamber, where it intersected with the monochromatic photon beam.

An elliptically polarizing undulator was adjusted to generate vertically polarized light, which optimized signal intensity for the vertically aligned electron analyzer (SCIENTA R4000). A photon energy of 250 eV was chosen as a compromise between the high flux at low photon energies inherent of the FinEstBeAMS beamline, and unwanted scattering from the Cu 3d core levels at lower photon energies. Photoelectrons with kinetic energies around 160 eV were collected, and the kinetic energy per step was 0.2 eV. To achieve a time-resolved investigation of the NPs surface composition, an individual spectrum consisting of a single sweep was recorded for 83 seconds, followed by a retracing time of 50 seconds. In total, one spectrum took 133 seconds (2 min 13 seconds), and this is therefore the time resolution. Also note that particle generation parameters were tuned (described in Section 2) to increase the signal for in-flight XPS.

The Zn 3p and Cu 3p core levels were probed, and their relative intensity was analyzed for different electrode compositions and over time. Data analysis and spectral fitting were conducted using CasaXPS software.^30^ Shirley backgrounds were subtracted from the XPS data. The Zn 3p doublets (3p_1/2_ and 3p_3/2_) were fitted using Gaussian/Lorentzian sum functions with an area ratio of 2 : 1 and a fixed spin–orbit splitting of 3.0 eV. The best fit was obtained using only the Zn^2+^ peaks, indicating that the surface contained ZnO. The FWHM was restricted to 2.8 eV for the Zn^2+^. The Cu 3p doublets (3p_1/2_ and 3p_3/2_) were fitted using Gaussian/Lorentzian sum functions with an area ratio of 2 : 1 and a fixed spin–orbit splitting of 2.45 eV. Shake-up satellite features characteristic of Cu^2+^, was included at 8 eV higher binding energy, indicating that the surface contained CuO.^31^

## 3 Results

### 3.1 Nanoparticle characteristics

**Fig. 1:**
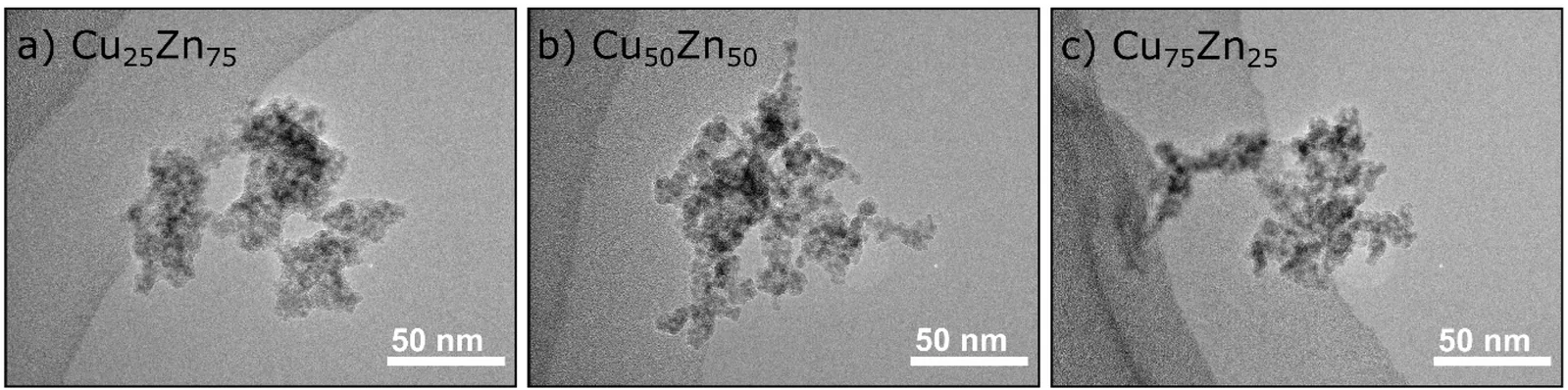
TEM images of CuZn NPs generated and deposited 10 min after the start of sparking using alloyed electrodes with compositions (a) Cu_25_Zn_75_, (b) Cu_50_Zn_50_, and (c) Cu_75_Zn_25_.

Size-selected CuZn particles with an electrical mobility diameter of 70 nm, were deposited after 10 minutes of sparking and studied using TEM. Fig. 1 shows representative particles generated from alloyed electrodes. Additional overview TEM images showing several agglomerates at lower magnification are provided in Fig. S2. The generated particles typically consist of agglomerates of smaller primary particles. It should be noted that the samples were exposed to ambient atmosphere prior to TEM analysis, and surface oxidation is therefore likely to have occurred post-synthesis. Qualitative inspection of the TEM samples did not reveal obvious differences in morphology, such as primary particle size or degree of coalescence, between particles generated using different electrode compositions. However, no statistical TEM-based morphology analysis was performed. Full size distributions of CuZn agglomerates generated in Ar using a charging current of 2 mA are shown in Fig. S3, with typical electrical mobility diameters ranging from 10 to 200 nm.

**Fig. 2:**
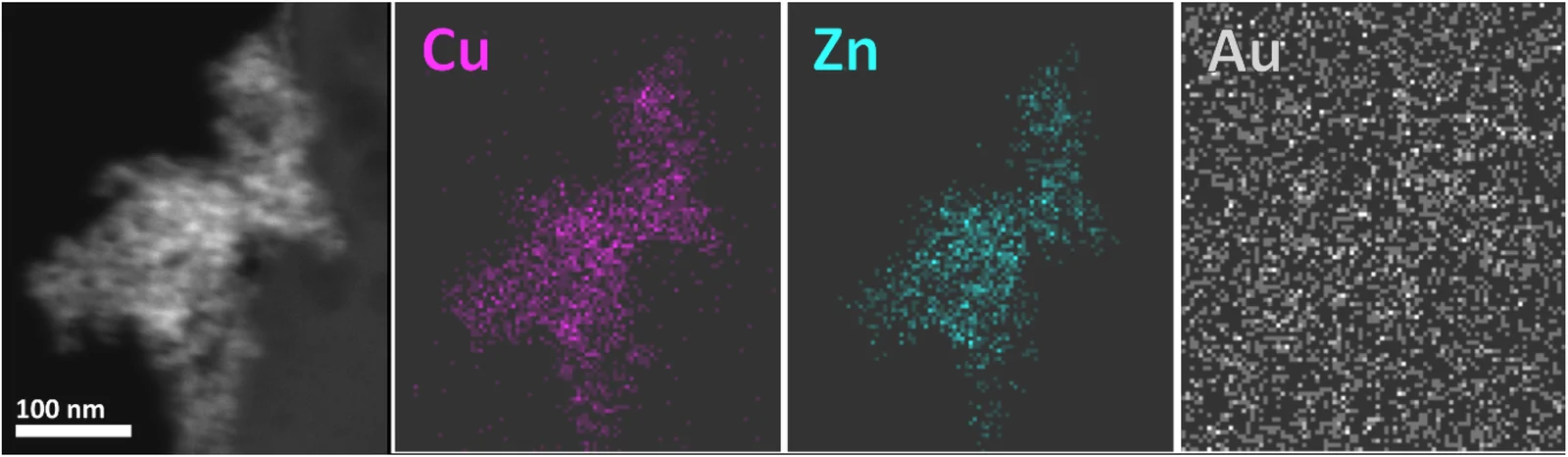
STEM image of a CuZn nanoparticle generated using the Cu_50_Zn_50_ electrodes. The STEM-EDS maps show the spatial distribution of Cu (magenta), Zn (cyan), and Au (white). The distribution of the Cu and Zn signals over the entire particle indicate an even elemental distribution.

The spatial distribution of Cu and Zn from a particle generated using Cu_50_Zn_50_ electrodes was investigated *via* STEM-EDS, see Fig. 2. The STEM-EDS maps demonstrate a homogenous spatial distribution of Cu and Zn throughout the entire particle, consistent with the formation of a homogeneous mixture of Cu and Zn.

**Fig. 3:**
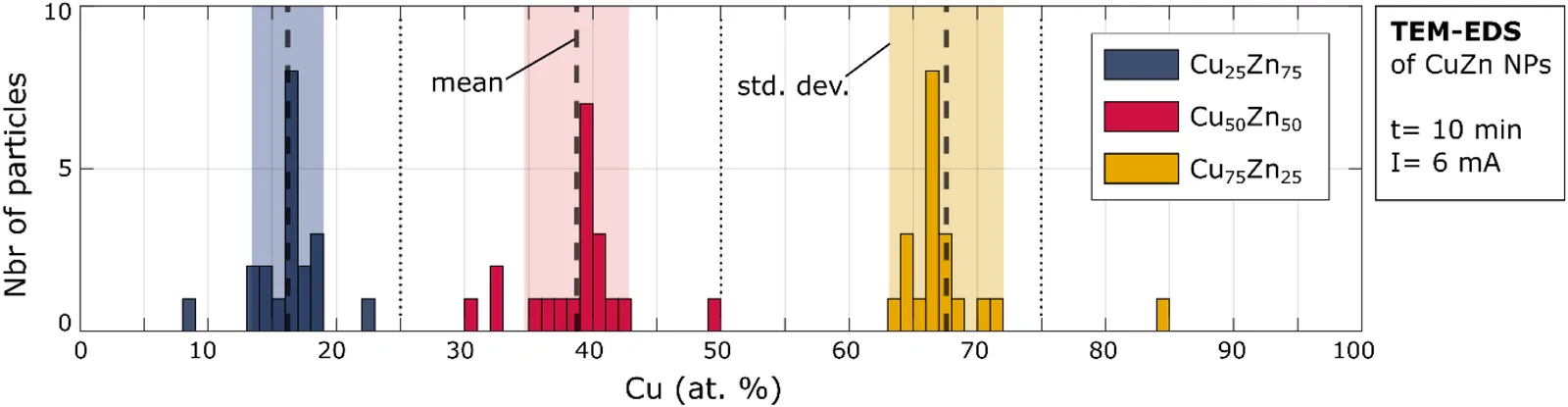
Composition of CuZn NPs determined by TEM-EDS analysis of individual particles. NPs were generated using a charging current of 6 mA and alloyed electrodes of Cu_25_Zn_75_ (dark blue), Cu_50_Zn_50_ (magenta), and Cu_75_Zn_25_ (yellow). The mean composition is indicated by dashed lines together with shaded regions representing ±1 standard deviation. The specified electrode compositions are indicated by dotted lines.

The composition of 20 individual particles was measured using TEM-EDS and the results are shown in Fig. 3. The mean composition of Cu with respect to Zn from each sample was 16.2, 38.8, and 67.6 atomic percent Cu, corresponding to a difference of −8.8, −11.2, and −7.4 atomic percentage points (at. pp.) from the specified electrode composition (dotted lines in the figure). The standard deviations were 2.8, 4.1 and 4.5 at. pp, in line with previously reported particle-to-particle compositional variations of NPs produced *via* spark ablation.^11^

The TEM-EDS results indicate that the nanoparticle composition deviates from the specified electrode compositions. To investigate how the surface of the electrodes used in this study is affected by sparking, it is analyzed using SEM and SEM-EDS.

### 3.2 Characterization of feedstock surface

The electrode surface of alloyed electrode pairs was investigated using SEM after 30 minutes of sparking using a charging current of 6 mA. Fig. S4 shows the surface structure with craters where the sparks hit the electrode, indications of melting, and a greatly varying surface morphology over the electrode surface. Erosion craters are estimated to be up to ∼10 µm in diameter, though only ∼1 µm deep. Based only on the surface structure from the SEM-images, it is difficult to observe any consistent differences among the electrode pairs. Instead, the surface composition was investigated.

**Fig. 4:**
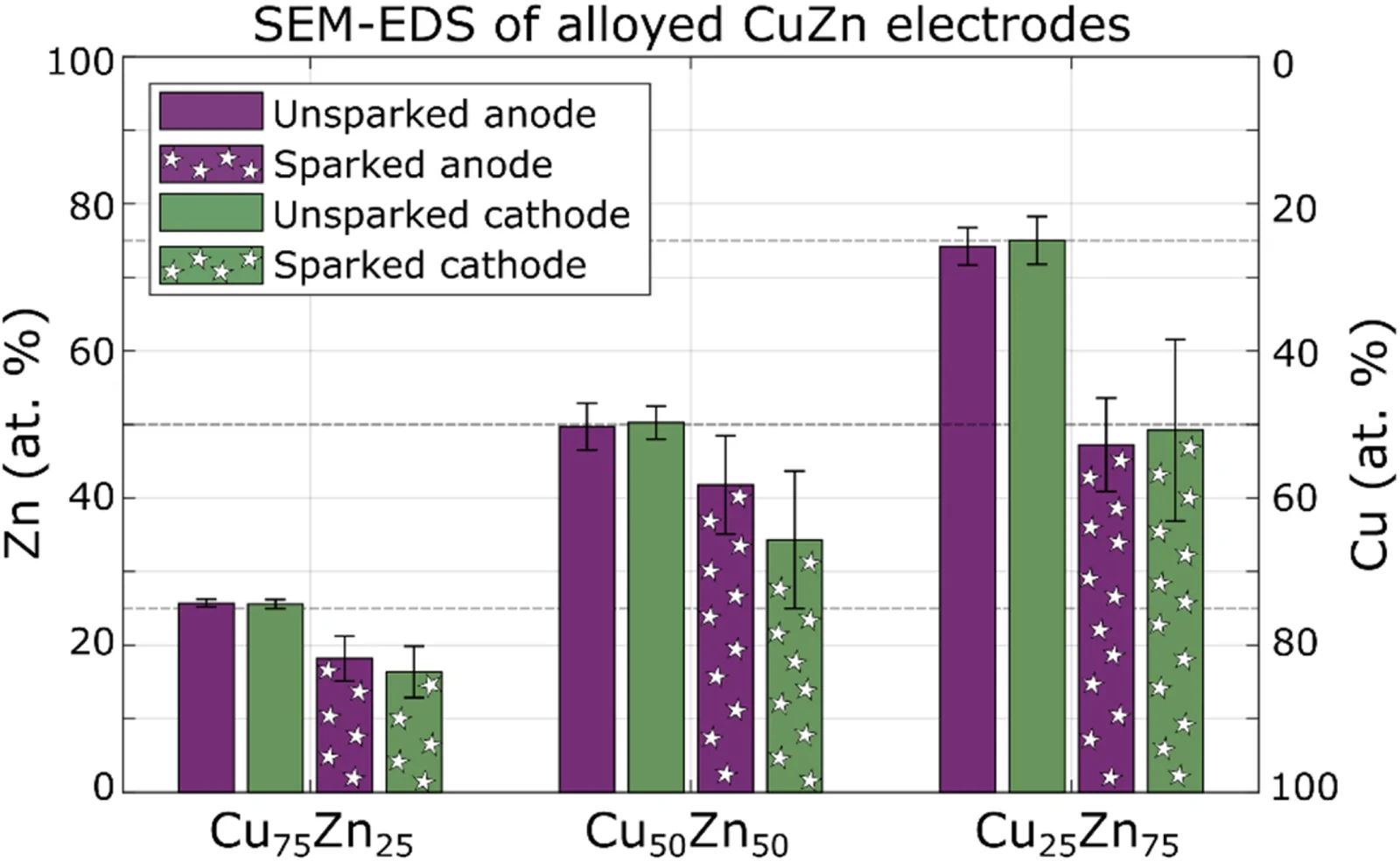
Average composition of the electrode surface obtained from 30 SEM-EDS point spectra of the anode (purple) and cathode (green). Error bars correspond to ±1 standard deviation. Stars indicate that the electrodes have been sparked for 30 minutes. The specified composition is indicated by dotted lines.

The composition of the CuZn alloy electrodes was analyzed by SEM-EDS before sparking (unsparked) and after approximately 30 min of continuous nanoparticle generation in Ar (sparked). For each electrode, 30 individual point spectra were collected from areas randomly distributed over the electrode surface, and the average Zn composition (relative to Cu) together with the corresponding standard deviation is shown in Fig. 4. Prior to sparking, the measured compositions of the alloyed electrodes agree well with the nominal values and standard deviations below 3.3 atomic percentage points. This verification was performed on polished electrode surfaces on two separate occasions. Between the measurements, the electrodes had been sparked and subsequently repolished, exposing material from below the original surface. The repeated agreement with the nominal compositions supports that the alloy composition is homogeneous.

The SEM-EDS results indicate that after sparking, there is a lower amount of Zn on the feedstock surface compared to before sparking. The average Zn composition after sparking corresponds to Zn losses of −7.5 and −9.2, −7.9 and −15.9, and −27.0 and −25.8 atomic percentage points, for anode and cathode electrodes with increasing nominal Zn content, respectively. Notably, the quite large change in composition is detected even though SEM-EDS has a relatively large interaction volume, extending several µm into the material (for the acceleration voltage used here). Consequently, the compositional differences must be large, and most probably extend beyond the immediate surface. The fact that clear differences are observed after only 30 minutes of nanoparticle generation indicates that sparking induces significant compositional modifications.

A SEM-EDS map on the Cu_50_Zn_50_ cathode and a line scan on the Cu_25_Zn_75_ anode after 30 minutes of sparking are shown in Fig. S5 and S6, respectively. The spatial distribution of Cu and Zn signal on the electrode surface indicate that local variations in composition exist, also indicated by the standard deviations between sparked and unsparked electrodes shown in Fig. 4. The electrodes were analyzed also after 5 h of sparking, showing that the average composition of the surface is similar to that after 30 minutes (see Fig. S7).

At this point, it is clear that particles generated 10 minutes after the start of sparking have a higher Zn content compared to the original composition of the electrodes. Furthermore, after 30 minutes of sparking, the electrode surface has a significantly lower average Zn content compared to the nominal values, indicating that the composition during extended sparking times might evolve over time.

### 3.3 Time-resolved XRF

**Fig. 5:**
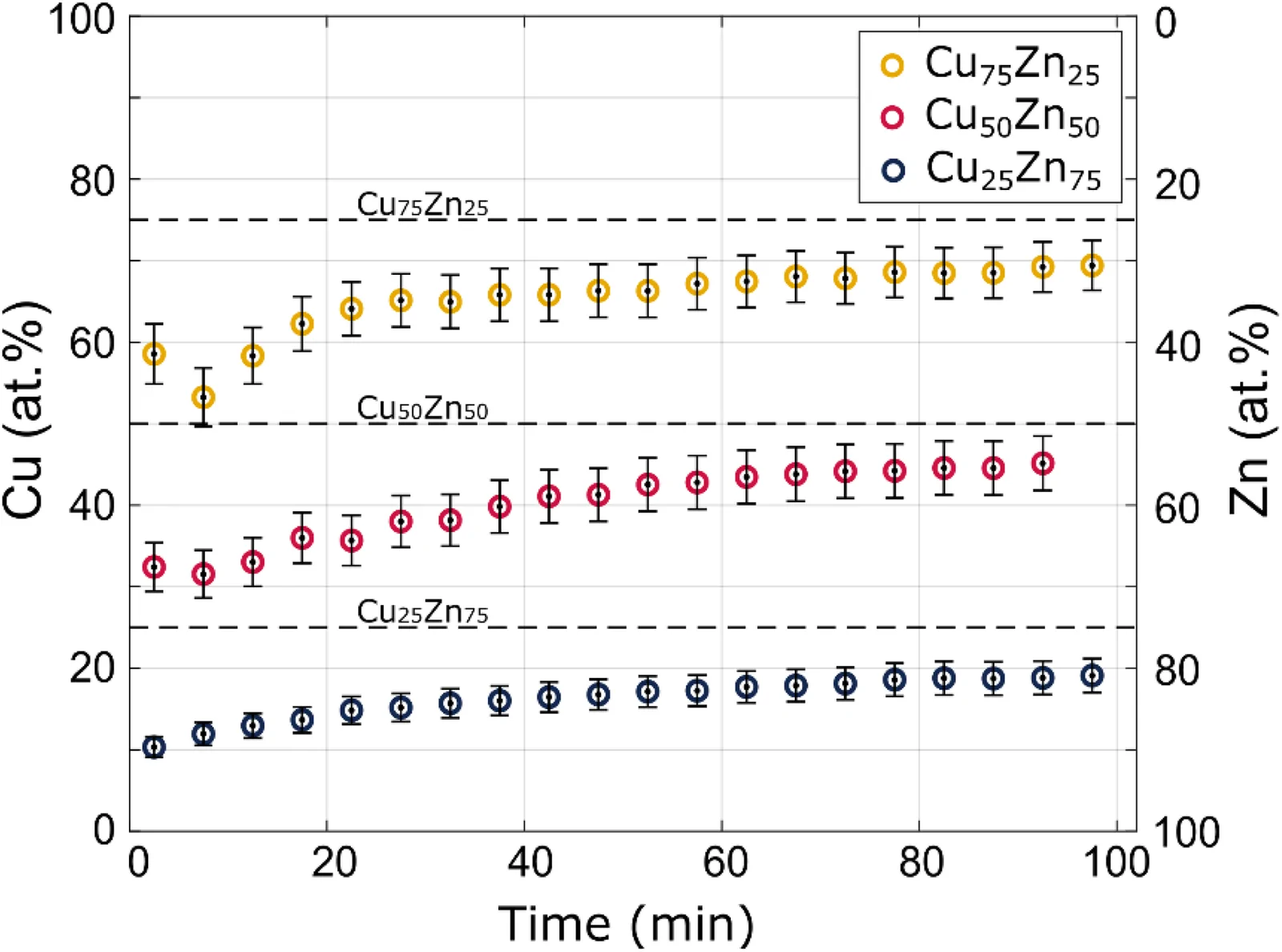
Time-evolution of the composition (from XRF) of freshly produced CuZn NPs. The original electrode compositions are included as black dotted lines in the figure. The average composition of particles produced from different CuZn alloys is indicated as follows; Cu_75_Zn_25_ (yellow), Cu_50_Zn_50_ (magenta), Cu_25_Zn_75_ (dark blue).

Time-resolved techniques are employed to obtain quantitative and time-resolved information of the nanoparticle composition, starting with analyzing the average composition of deposited NPs *via* XRF. The Lund spark ablation setup (run with 2 mA charging current) was coupled to an Xact instrument capable of measuring the composition of NPs deposited on a filter with a temporal resolution of 5 minutes. Elemental analysis was performed directly after each 5-minutes particle collection interval using XRF. The results when the three different alloyed electrodes are used to produce particles for about 100 minutes are presented in Fig. 5. For reference, the specified electrode composition is added as dotted black lines, while the measured particle composition is represented by yellow (Cu_75_Zn_25_), magenta (Cu_50_Zn_50_), or dark blue (Cu_25_Zn_75_) circles with measurement uncertainty added as error bars.

According to the XRF results presented in Fig. 5, it is clear that the nanoparticle composition does not correspond to the specified electrode composition, and that it changes over time. For all electrodes, the particle composition has a lower Cu content compared to that of the specified feedstock, but over time it seems to increase and approaches the specified composition. The first measurement point corresponding to particles produced during the first 5 minutes of sparking shows a Cu difference of about −15 atomic percentage points from the nominal composition, while after 90 minutes, the difference is only about −5 at. pp. For reference, the AgAu material system was tested (see Fig. S8) in the same manner as the CuZn but did not show a similar change in nanoparticle composition over time.

**Fig. 6:**
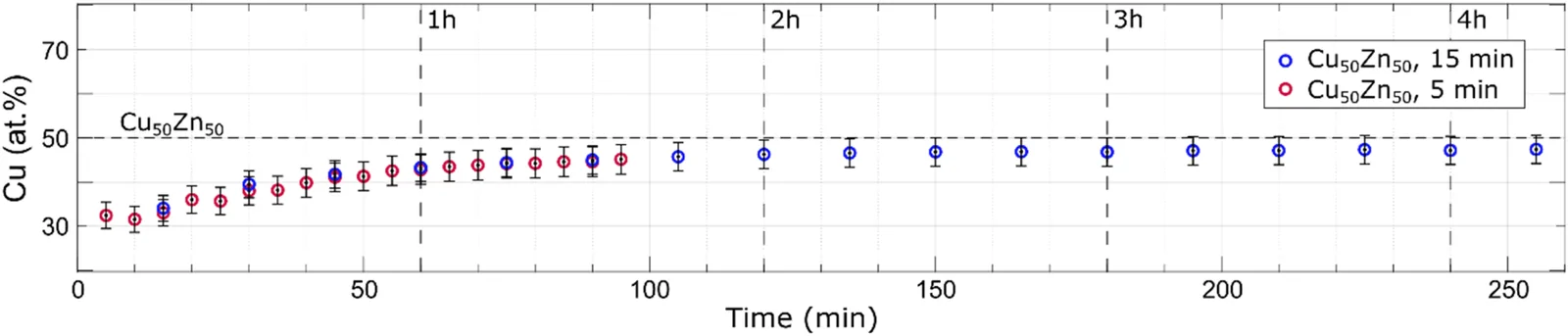
The average composition measured by XRF of CuZn NPs generated over ∼4 hours. The data points represent the average composition of particle samples deposited and analyzed for 15 minutes (blue). For comparison, the 5-minutes interval measurements from the same electrodes are also included (magenta). The dashed line indicates the original electrode composition.

A longer measurement and investigation of the nanoparticle composition from the Cu_50_Zn_50_ electrode at 15-minutes intervals can be seen in Fig. 6 together with the 5-minutes data from Fig. 5. The 15-minutes measurements match well with the 5-minutes data, showing reproducibility of the results. Over time, the average nanoparticle composition slowly approaches that of the originally specified electrode composition, and after 4 hours, it differs by only about 2.5 at. pp. This result shows that, for the Cu_50_Zn_50_ electrode, the specified composition will be reached if the sparking is done long enough. However, since no investigations were performed for even longer sparking times, it is not known if the composition continues to drift, above the specified one.

The nominal electrode composition is neither preserved on the electrode surface nor in the resulting particles. The particle composition is changing with sparking time, though it seems to approach the nominal composition with time. The electrode surface composition indicates Zn depletion, as compared to the nominal Zn content. However, the nanoparticle composition depends on two elements, Cu and Zn, so the composition can change due to a variation of one of them or both. Furthermore, it is not yet known if the change in composition happens already during the particle formation stage or if it changes later in the setup. Therefore, the particle formation stage is investigated by measuring light emission from the plasma including signals from ablated and excited Zn and Cu atoms.

### 3.4 Time-resolved plasma OES

**Fig. 7:**
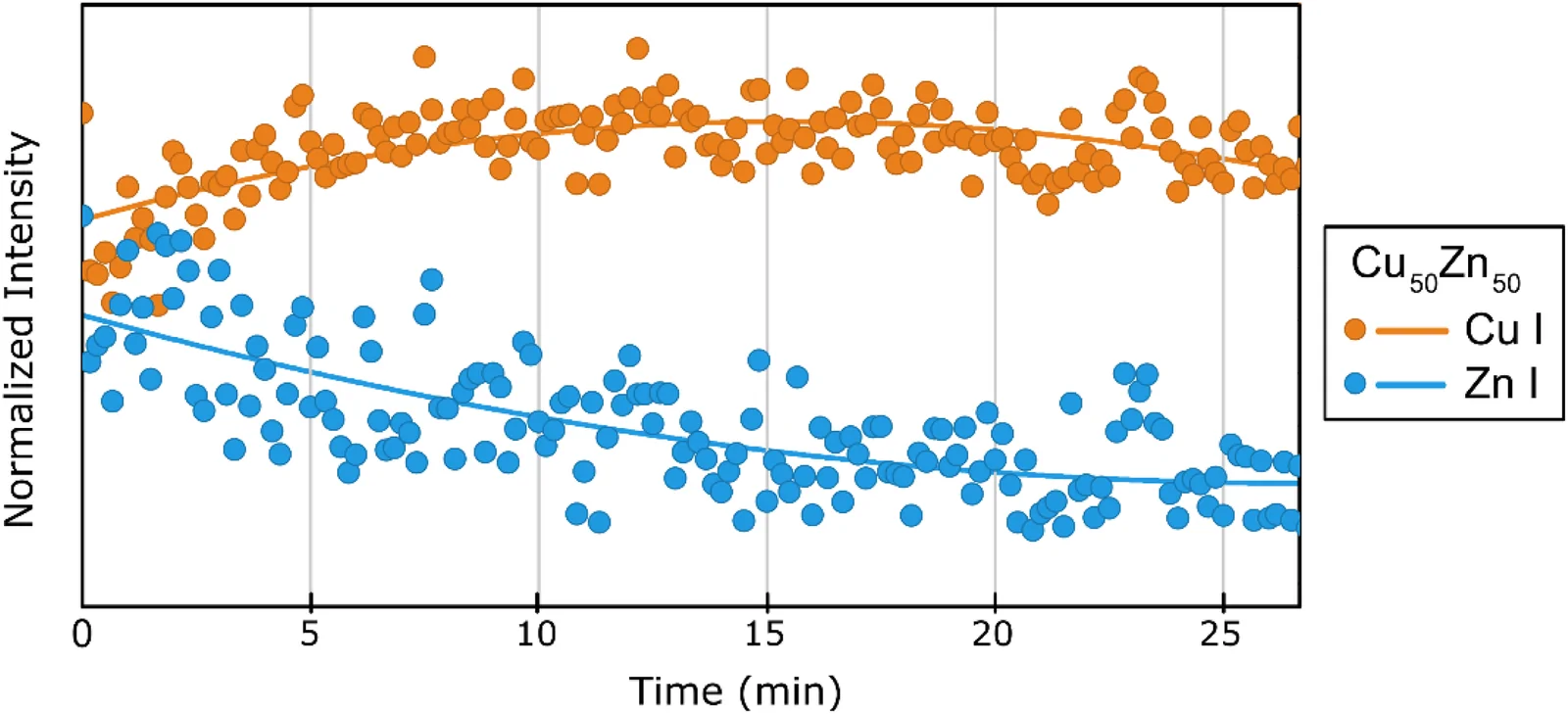
Integrated optical emission signals from Cu and Zn over 25 min of spark ablation. The Cu (orange) and Zn (blue) emission intensities are normalized to the Ar carrier gas signal.

Fig. 7 shows the intensity (normalized to Ar) for the selected Cu I and Zn I atomic lines from the OES over time, for spark discharge generation of Cu_50_Zn_50_ electrodes with a charging current of 6 mA in Ar. The intensity signals from OES qualitatively show the excited Cu and Zn atomic species present in the plasma during particle generation. The results indicate that the amount of Zn – assumed to be proportional to the normalized Zn I intensity – decreases as time progress. Similarly, the amount of Cu is assumed to be proportional to the corresponding emission intensity – here shown to increase with time. Note that the results presented here cannot provide direct quantitative information of the Cu–Zn ratio but shows the relative evolution over time. However, a qualitative estimation can be seen in Fig. S9, which shows how the intensity of Cu compared to the total intensity from Cu and Zn evolve over time. The results indicate an increase of 10–20% in the Cu “content” of the total signal, which is in line with the increase in average Cu content of NPs over time seen from XRF.

From the OES results, it seems that the change in relative Cu–Zn composition is not only due to a decrease in the Zn ablated over time, but also due to an increase in the Cu ablated over time, and together this results in the observed composition variation. Furthermore, OES only detects signal from excited species, and so might not represent the concentration ratio of the whole population. The signal from ICP-MS does not discriminate in this sense, and the following analysis is therefore continued with in-flight, time-resolved ICP-MS.

### 3.5 Time-resolved ICP-MS

**Fig. 8:**
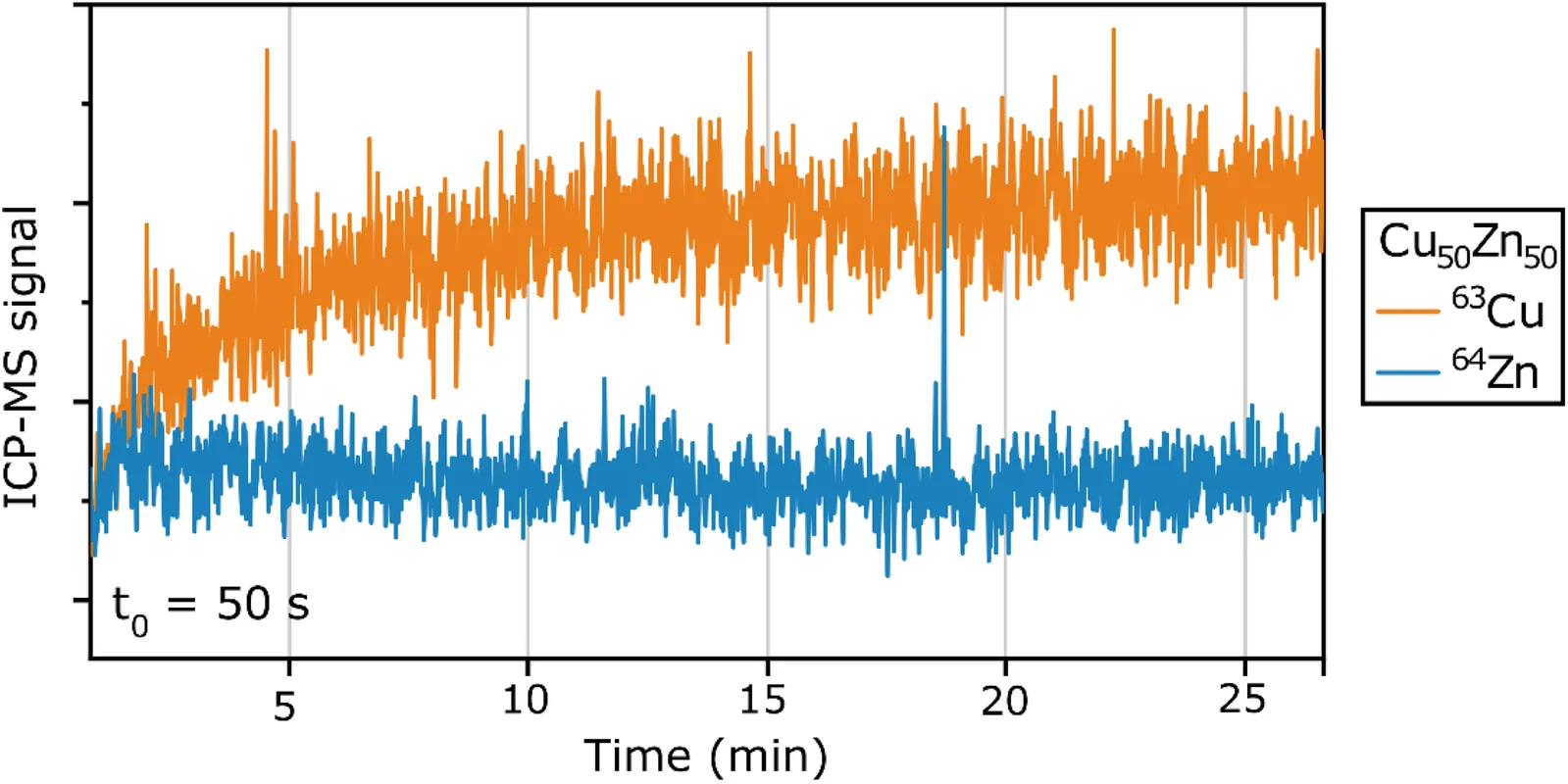
Time-resolved ICP-MS signals from the ^63^Cu (orange) and ^64^Zn (blue) isotopes during nanoparticle production using Cu_50_Zn_50_ alloyed electrodes in Ar and a charging current of 6 mA. A delay of ∼50 seconds reflects the particle residence time before reaching the ICP-MS plasma. The signals are corrected for variations in particle number concentration.

To gain further qualitative understanding of how the relative fraction of Cu and Zn in the NPs changes over time, and with a higher time resolution than XRF, the Clausthal spark ablation setup was connected to an ICP-MS to allow for in-flight and time-resolved analysis of the produced NPs. The ICP-MS setup monitors how a specific elemental signal from the produced NPs changes over time. The result from the Cu_50_Zn_50_ electrodes sparked in Ar using a charging current of 6 mA over a period of about 25 minutes is shown in Fig. 8 below. While the Zn signal (blue) shows a slight decrease over time, the Cu signal (orange) exhibits an increase, in line with the OES results above.

Though the signals from ICP-MS cannot directly be translated to a composition, the intensity from Cu was compared to the total intensity of Cu and Zn in Fig. S10, providing an indication of the relative composition of the particles. The trend of the relative Cu composition is like that from OES in Fig. S9, confirming that the change in composition is both due to a decrease in Zn ablation and at the same time an increase in the Cu ablation.

### 3.6 In-flight XPS

The techniques used up until now have only been able to give information of the nanoparticle bulk composition (TEM-EDS, XRF, ICP-MS). However, for most applications it is the surface of the particles that is of greatest interest. Therefore, in-flight XPS is applied to the particles to find out information of their surface composition over time.

Time-resolved in-flight XPS measurements were performed at a synchrotron facility. CuZn NPs were generated from Cu_50_Zn_50_ alloyed electrodes. A limitation of the in-flight XPS measurements was that a sufficiently high signal could not be obtained when Ar was used as carrier gas, probably due to a generally lower particle production in Ar compared to N_2_-based gases.^32^ Therefore, N_2_ was used as carrier gas, to obtain a signal allowing for time-resolved analysis of the particles in-flight. In addition, the charging current was increased to 30 mA to further enhance the particle output.

**Fig. 9:**
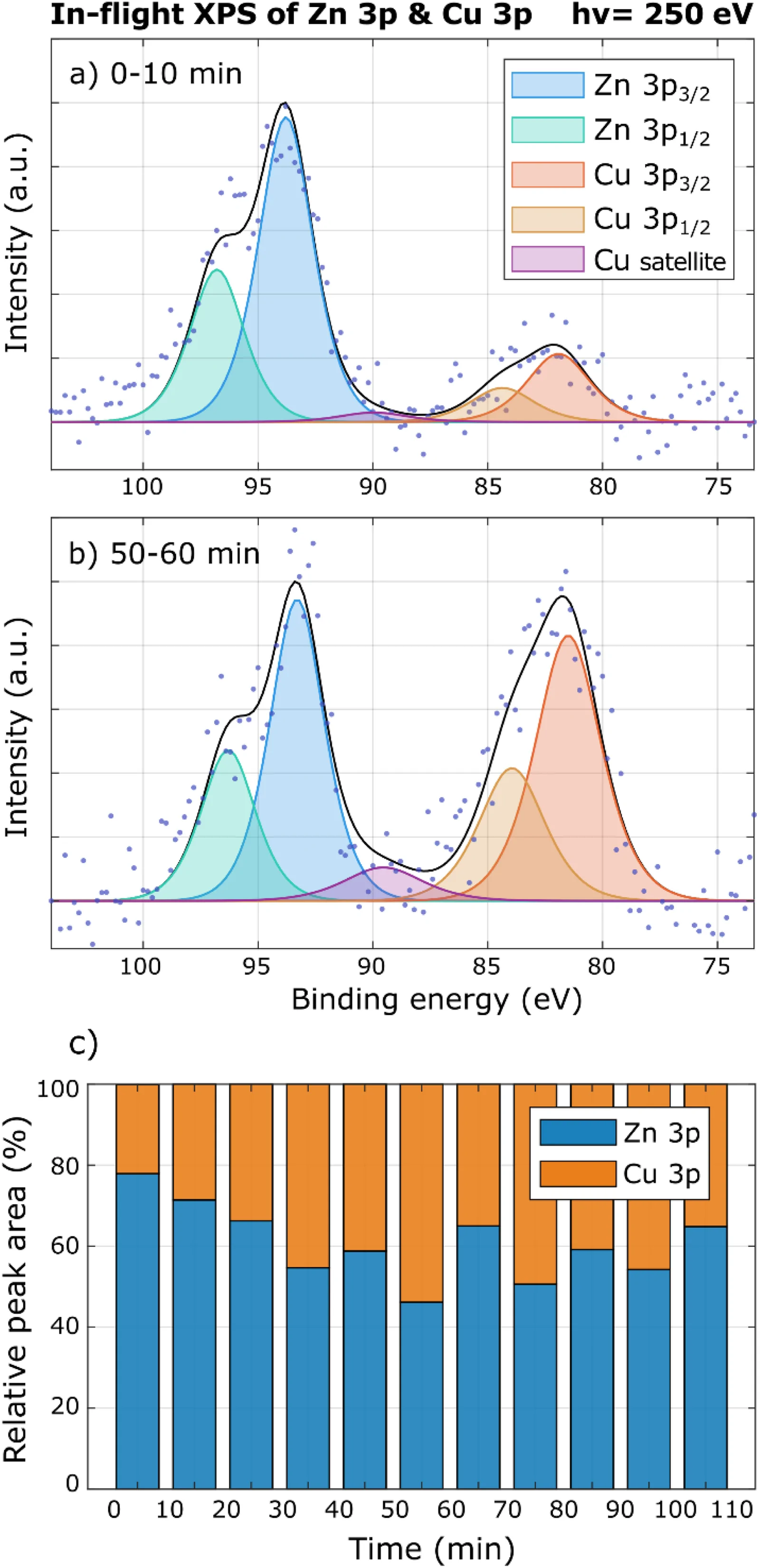
In-flight XPS of the Zn 3p and Cu 3p core levels from NPs generated in N_2_ using Cu_50_Zn_50_ electrodes and a charging current of 30 mA. (a) Spectra acquired during the first 10 minutes of nanoparticle generation. (b) Spectra acquired between 50 and 60 min after the start of generation. Note that the spectra are normalized against the Zn 3p peak intensity. (c) Time evolution of the relative Zn and Cu 3p peak areas.

Fig. 9a and b show the resulting photoelectron spectra, including both Zn 3p and Cu 3p, acquired during the first 10 minutes and between 50 and 60 min after the start of nanoparticle generation, respectively. Comparing the spectra, a clear change in the relative surface composition of Zn and Cu is observed (note that the spectra are normalized against the Zn 3p peak intensity). The XPS spectra reveal that the surface contains ZnO and CuO, and while ZnO dominates the surface of the particles generated the first 10 minutes, CuO increases over time. Previous studies of CuZn NPs have shown that Zn is preferentially in the form of ZnO on the nanoparticle surface, but that the oxidation state it is dependent on the environment being oxidative or reductive as well as on the temperature.^1,27^

Fig. 9c shows the evolution of the relative Zn 3p and Cu 3p peak areas over time, from start to ∼2 hours of sparking. The corresponding fitted peak areas before normalization are shown in Fig. S11. While these values cannot be directly converted into an absolute atomic composition of the surface, they provide a qualitative indication of compositional changes at the nanoparticle surface. The trend of decreasing Zn relative to Cu from time-resolved XPS is consistent with the results obtained using the other time-resolved characterization techniques reported in this study (OES, ICP-MS).

## 4 Discussion

The present study shows that continuous sparking of alloyed CuZn electrodes modifies the electrode surface, leading to a time-dependent evolution of both the surface composition and the composition of the generated nanoparticles. In contrast to previously studied alloy systems such as Ag–Au, where the nanoparticles composition closely followed the feedstock composition,^14^ Cu–Zn shows behavior consistent with a distillation-like effect. This is likely related to the large difference in vapor pressure between Cu and Zn, together with the much lower melting point of Zn (see Fig. S12 showing data from ref. 33).

The SEM-EDS, XRF, OES, ICP-MS, and XPS results consistently indicate preferential Zn removal during sparking. Together, these techniques probe different stages of the process: SEM-EDS reflects the modified electrode surface, XRF and ICP-MS probe the particle composition, OES probes the plasma phase during ablation, and in-flight XPS gives information on the nanoparticle surface. For example, after 30 min of sparking with the Cu_50_Zn_50_ electrodes, the electrode surface contained approximately 40 at% Zn, whereas the generated nanoparticles contained approximately 60 at% Zn. Time-resolved OES and ICP-MS further showed decreasing Zn signals and increasing Cu signals during the first 30 min, confirming a compositional change during sparking. These observations indicate that Zn is preferentially ablated or evaporated from the electrode surface, gradually leaving behind a Cu-enriched surface.

A Zn-rich native oxide layer on the electrodes may contribute to the initial rapid change in composition, but it cannot explain the longer evolution observed over tens of minutes to hours, since such a thin surface layer would be removed early during sparking. Likewise, diffusion may contribute locally in transient molten regions, but estimated diffusion lengths are small compared with the micron-scale erosion craters formed during spark ablation, and the average electrode surface temperature is expected to remain below 100 °C^34^ (see diffusion and electrode temperature discussion in SI). Local transient melting and resolidification during repeated spark impacts may nevertheless promote near-surface reorganization. Therefore, long-range diffusion from the bulk is unlikely to be the primary source of Zn replenishment.

**Fig. 10:**
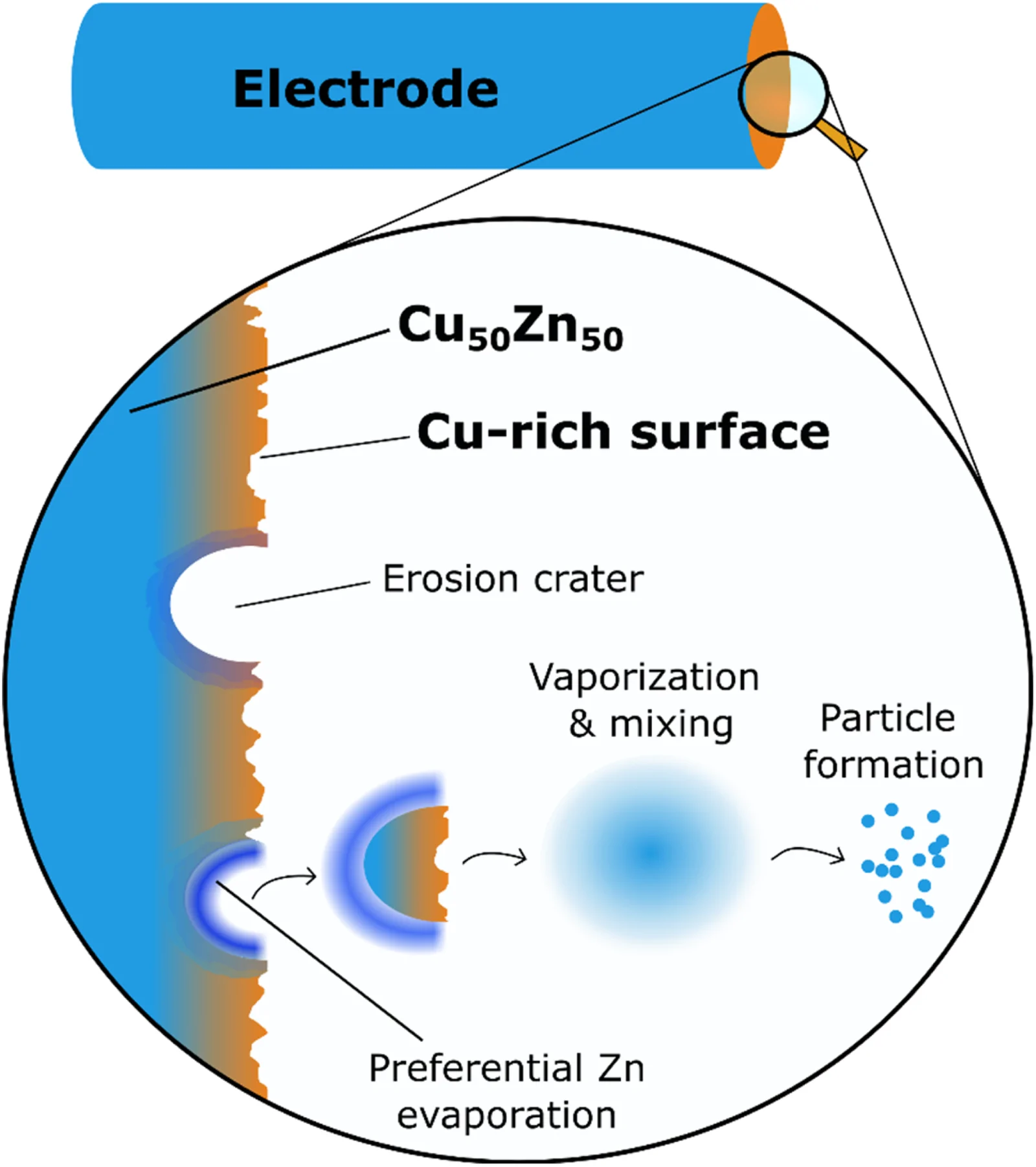
Schematic illustration of an alloyed Cu_50_Zn_50_ electrode surface when steady state is achieved (proposed hypothesis based on results in this study). Over time, the surface becomes enriched in Cu due to preferential Zn evaporation. Spark erosion craters can reach deeper into the electrode surface, hence sampling both Cu-rich surface and “fresh” alloy electrode material, ensuring a constant supply of new Zn. The ablated material is vaporized and mixed, leading to well-mixed CuZn NPs.

The fact that polishing the electrode surface using sandpaper, which typically removes tens of micrometers of material, restores the nominal alloy composition (confirmed by SEM-EDS) indicates that the depletion zone is confined to relatively shallow depths. Based on the results in this study, we propose a qualitative mechanism, in which preferential Zn evaporation leads to formation of a Cu-rich surface layer, while repeated spark erosion, redeposition, and exposure of less-depleted alloy material provide a continued supply of Zn. The ablated material is subsequently vaporized and mixed in the gas phase, resulting in well-mixed CuZn NPs. This proposed mechanism is illustrated schematically in Fig. 10 and should be regarded as a hypothesized mechanism rather than a direct structural reconstruction of the electrode surface.

A simple semi-quantitative ablation model was developed to test whether preferential Zn evaporation combined with effective surface renewal can reproduce the temporal CuZn NP composition trends observed in this study. The model/fit reproduces the general trends observed by time-resolved XRF, as shown in Fig. S13 and discussed in the SI. Importantly, the predicted steady-state nanoparticle composition does not necessarily coincide with the nominal feedstock composition. The relatively slow change in composition over time may be related to the fact that approximately 99.5% of the ablated material is known to redeposit on the electrode surfaces, while only ∼0.5% contributes to nanoparticle formation.^19^

To reproduce the long-time Cu_50_Zn_50_ XRF data using the model, the effective surface-renewal parameter *β* had to exceed the parameter describing material ablation α. Physically, this suggests that each spark modifies a larger near-surface volume than the fraction of material ultimately released as gas-phase nanoparticles. Preferential Zn evaporation continuously enriches the uppermost electrode surface in Cu, while repeated spark erosion, local melting, redeposition, and exposure of partially fresh alloy provide a continuous supply of Zn. The system therefore approaches a stable particle composition close to, but not necessarily identical to, the original electrode composition.

Overall, the observed behavior likely reflects an interplay between preferential Zn evaporation, redeposition and inter-electrode material transfer, local melting and resolidification, and continuous replenishment of Zn through partial renewal of the electrode surface. Although further investigation is needed to fully resolve the underlying mechanisms, the model provides a qualitative explanation for the gradual compositional shift observed during continuous spark ablation of CuZn alloys.

## 5 Conclusion

This study demonstrates the gas-phase synthesis of bimetallic Cu–Zn NPs *via* spark ablation, showing that compositions across a wide Cu–Zn range can be accessed by selecting appropriate feedstock materials. Compared with wet chemical synthesis, spark ablation generally provides less precise control over particle size and morphology, but offers advantages in terms of composition control. In this work, internally homogeneous CuZn alloy nanoparticles were produced, and their composition was shown to evolve with sparking time before reaching a dynamic steady state. Thus, despite the substantial differences in thermophysical properties between Cu and Zn, stable and tunable nanoparticle compositions can be achieved.

A clear time dependence in nanoparticle composition was identified when starting from new “un-sparked” electrodes. This behavior was systematically investigated using complementary, time-resolved techniques probing (i) the electrode surface composition, (ii) the ensemble nanoparticle bulk and surface composition, (iii) the single-particle composition, and (iv) the plasma phase. Together, these measurements provide a comprehensive picture of the processes governing compositional evolution. The results indicate that prolonged sparking leads to a progressively stabilized nanoparticle composition, reflecting the establishment of a dynamic balance between preferential evaporation, redeposition, and electrode composition modification. Based on these observations, a qualitative mechanism, supported by a simple ablation model, is proposed to explain the temporal evolution of composition in CuZn spark ablation.

By combining advanced time-resolved characterization with controlled nanoparticle generation, this work provides critical insight into bimetallic nanoparticle formation in spark ablation. The findings contribute to refining the understanding of the process and establish guidelines for achieving controlled and tunable nanoparticle compositions. This capability is essential for tailoring material properties, for example in catalytic applications where activity and selectivity are strongly composition dependent.

## Author contributions

LJ and MEM conceived the study. LJ, VO, and AK contributed to the design of the study. LJ carried out time-resolved XRF measurements, SEM, SEM-EDS, TEM, TEM-EDS, STEM-EDS, data curation, formal analysis, visualization, and drafted the manuscript. LJ was also the principal investigator for the XPS experiments performed at MAX IV. VO was responsible for the ICP-MS measurements and contributed to investigation, methodology development, data curation, formal analysis, visualization, and interpretation of the results. AK was responsible for the OES measurements and contributed to the investigation, methodology development, formal analysis, software development, visualization, and interpretation of the results. DM, TK, CP, JR, and ACE contributed to the XPS experiments and analysis. ACE also provided access to the XRF instrumentation. CP additionally contributed to methodology development. ZG, KD, APW, AK, and MEM supervised the work and contributed to funding acquisition. All authors contributed to discussions, commented on the manuscript, and approved the final version.

## Conflicts of interest

There are no conflicts of interest to declare.

## Supplementary Material

NA-008-D6NA00440G-s001

## Data Availability

The data supporting this article have been included as part of the supplementary information (SI). Supplementary information is available. See DOI: https://doi.org/10.1039/d6na00440g.
