# Supplementary material for: Gas-phase synthesis and time-resolved composition analysis of CuZn nanoparticles
Source: Nanoscale Adv. 2026 Jul 3. Online ahead of print. doi: 10.1039/d6na00440g (PMC13353899; doi:10.1039/d6na00440g)
Supplement: NA-OLF-D6NA00440G-s001 [file NA-OLF-D6NA00440G-s001.pdf]

Supplementary Information (SI) for Nanoscale Advances.
This journal is © The Royal Society of Chemistry 2026

# Supplementary Information:

## Gas-phase synthesis and time-resolved composition analysis of CuZn nanoparticles

Linnéa Jönsson[1,2], Vinzent Olszok[3], Dániel Megyeri[4], Thomas Krinke[1,2], Calle Preger[2,5], Jenny Rissler[2,6], Axel. C. Eriksson[2,6], Zsolt Geretovszky[4], Knut Deppert[1,2], Alfred P. Weber[3], Attila Kohut[4], and Maria E. Messing[1,2,7]

[1]Solid State Physics, Lund University, 221 00, Lund, Sweden
[2]NanoLund, Lund University, 221 00, Lund, Sweden
[3]Clausthal University of Technology, Institute of Particle Technology, 38678, Clausthal-Zellerfeld, Germany
[4]Department of Optics and Quantum Electronics, University of Szeged, 6720, Szeged, Hungary
[5]MAX IV Laboratory, Lund University, Box 118, Lund 221 00, Sweden
[6]Ergonomics and Aerosol Technology, Lund University, 221 00, Lund, Sweden
[7]Quantum Device Physics Laboratory, Department of Microtechnology and Nanoscience, Chalmers University of Technology, 412 96, Gothenburg, Sweden

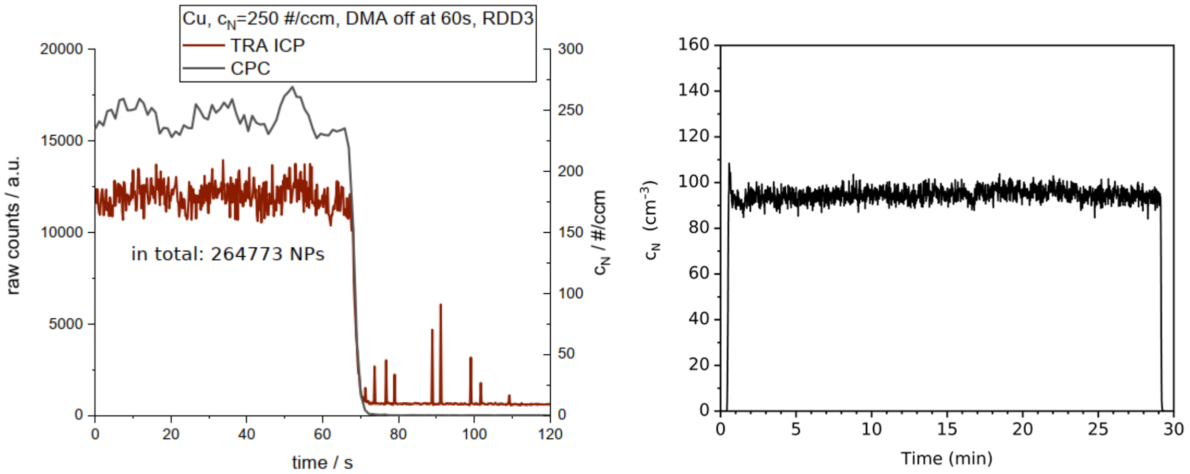


*Figure S1. Left: Particle concentration being injected into the ICP-MS over time, measured by a CPC. Right: Raw counts from time-resolved analysis (TRA) by ICP-MS over time, left axis, and concentration of particles measured by a CPC over time. After 60 seconds, the supply of particles was turned off, resulting in a drop in signal from both the ICP-MS and the CPC.*

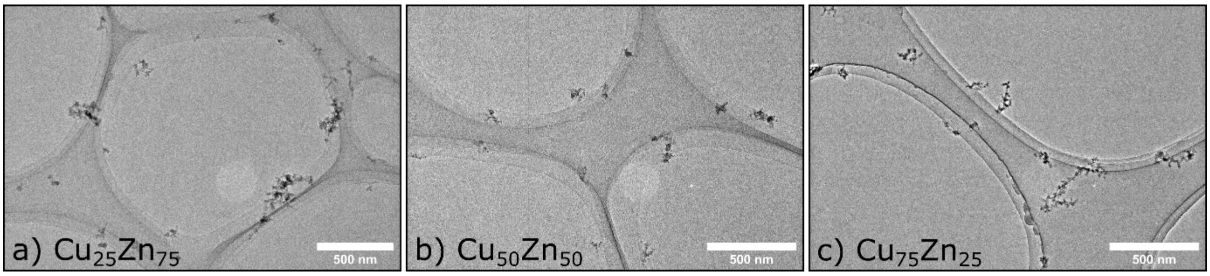


*Figure S2. Overview TEM images of CuZn nanoparticles generated from three different alloyed electrode pairs. The particles were mobility-selected at an electrical mobility diameter of 70 nm before deposition. The images show several agglomerates at lower magnification and support the qualitative assessment that no obvious morphology differences were observed between samples. No statistical TEM-based morphology analysis was performed.*

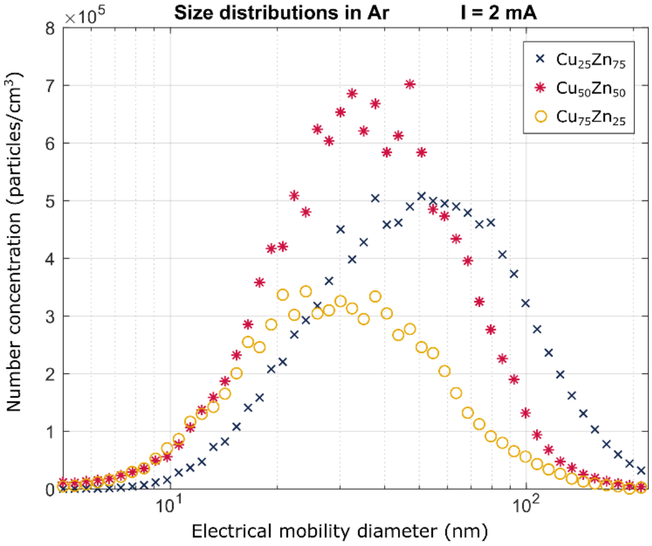


*Figure S3. Size distributions of CuZn particles generated in Ar with a charging current of 2 mA, from three different alloyed electrode pairs.*

Among the investigated compositions, the $Cu_{25}Zn_{75}$ feedstocks produce the largest particles, whereas the equiatomic $Cu_{50}Zn_{50}$ feedstocks yields the highest modal particle concentration by number. In contrast, the $Cu_{75}Zn_{25}$ feedstock results in the lowest modal number concentration and the smallest modal diameter. Note that the size distributions in Figure S3 should only be interpreted qualitatively, see Section 2.2.

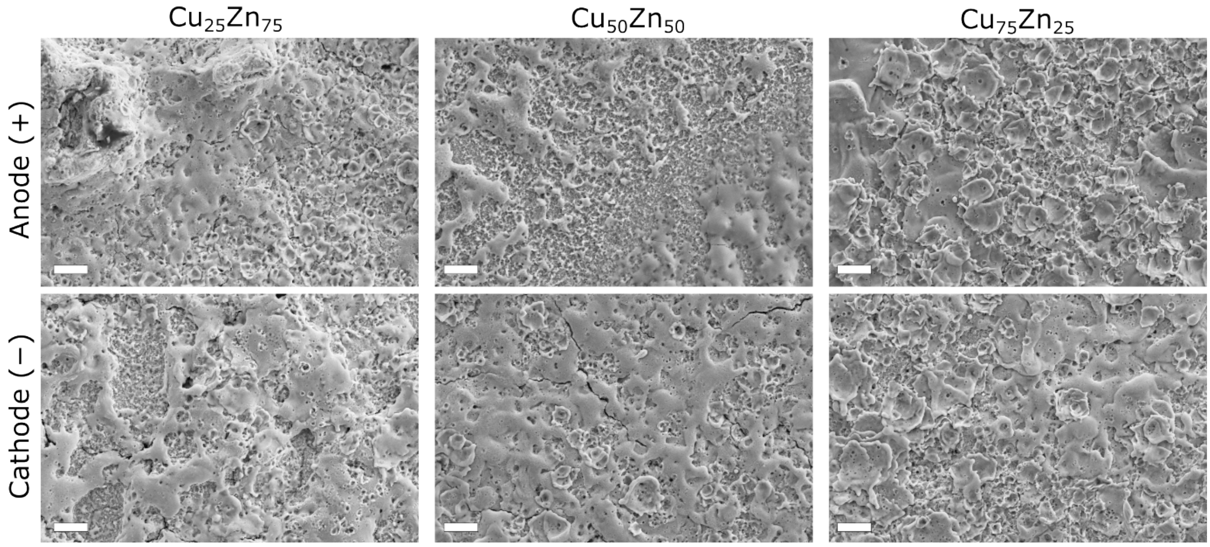


*Figure S4. SEM images of electrode surfaces after 30 minutes of sparking with 6 mA charging current in Ar. The top row shows the initially positive anode electrodes while the bottom row shows the initially negative electrodes. Scale bars are 10 µm.*

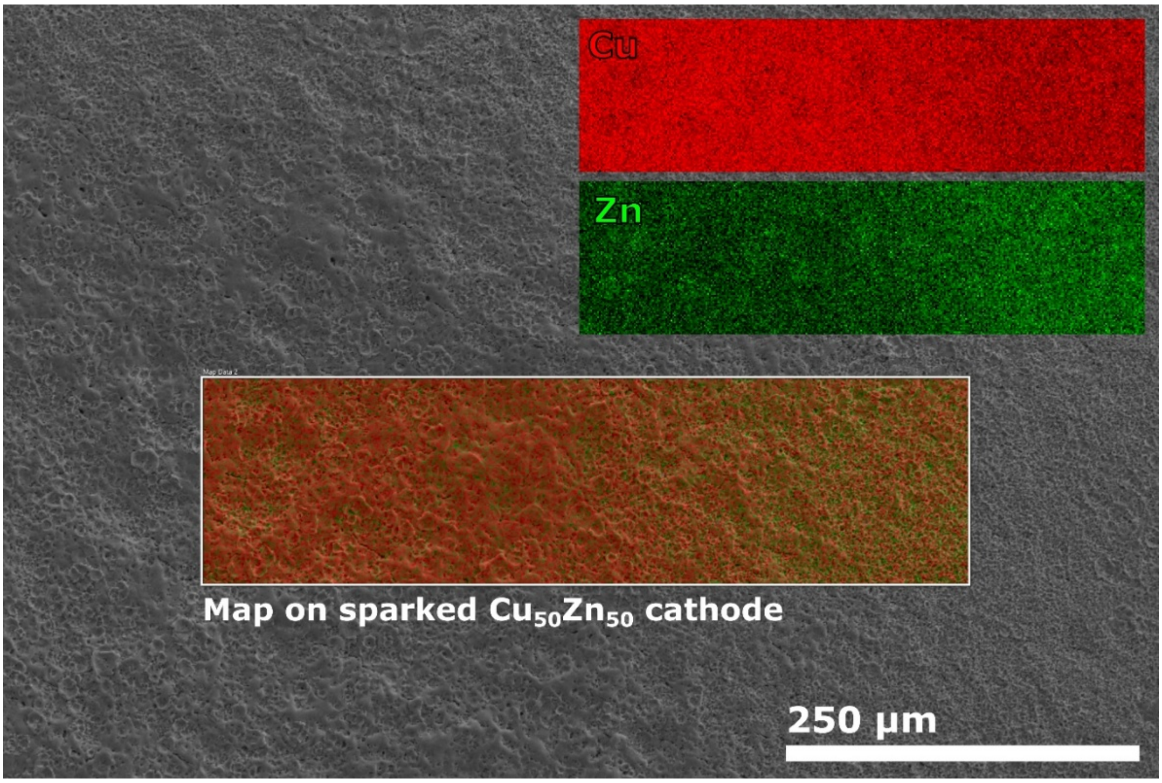


*Figure S5. SEM-EDS map on the surface of a sparked $Cu_{50}Zn_{50}$ cathode. The Zn signal seems to be higher (and the Cu signal lower) on the right-hand side of the map, indicating that the surface composition can be different on different parts of the electrode surface.*

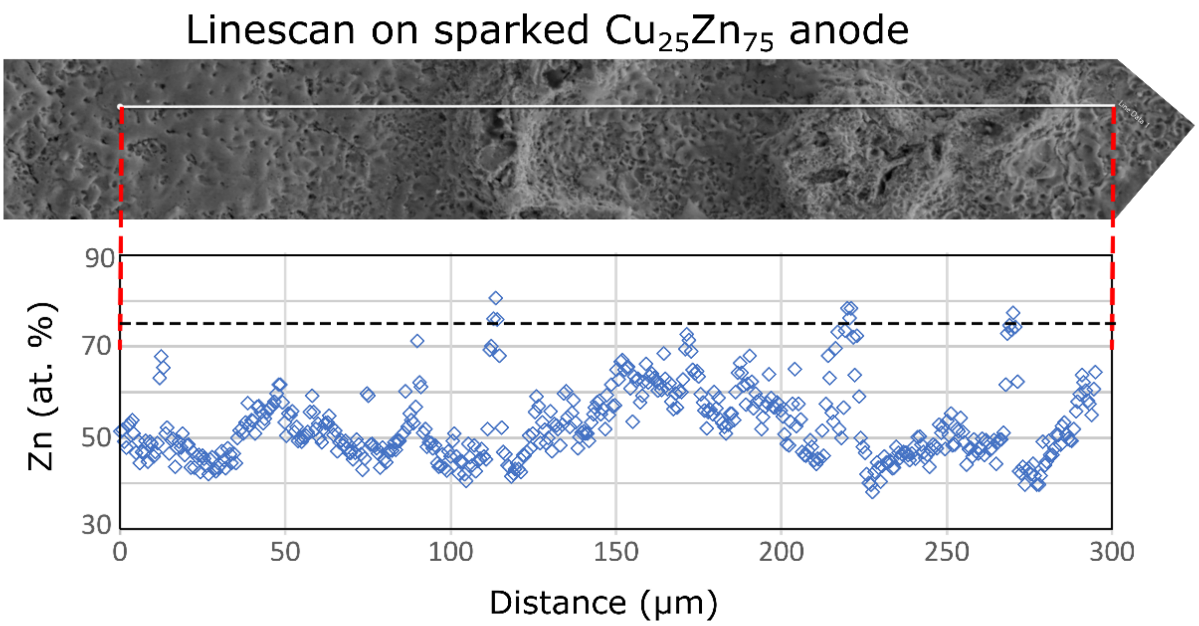


*Figure S6. SEM-EDS line scan of a sparked $Cu_{25}Zn_{75}$ anode. The specified composition is indicated by the dashed line. The Zn content is on average lower than the specified, though protrusions from the surface at approximately 115 µm, 220 µm, and 270 µm show higher Zn content.*

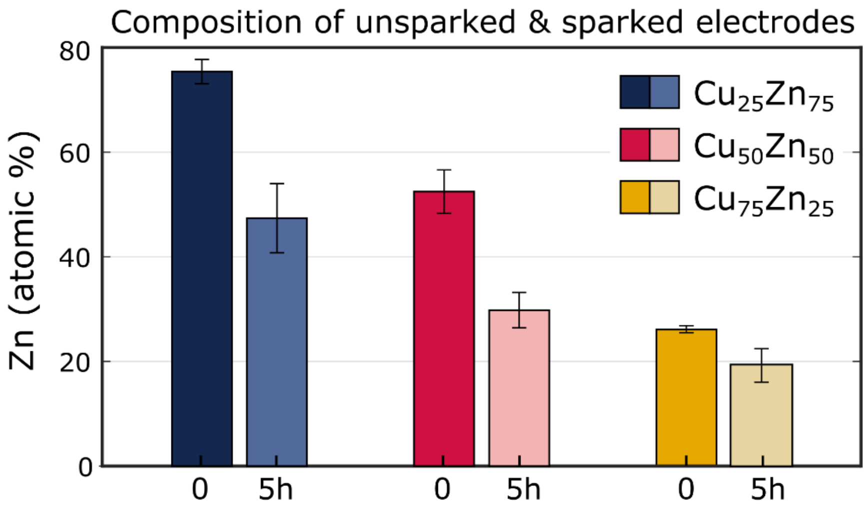


*Figure S7. Average composition of the electrode surface obtained from 30 SEM-EDS point spectra of an electrode before and after 5 h of sparking using a charging current of 6 mA in Ar. Note that the average composition after 5 h of sparking is similar to that after 30 min of sparking in Figure 4 of the main manuscript.*

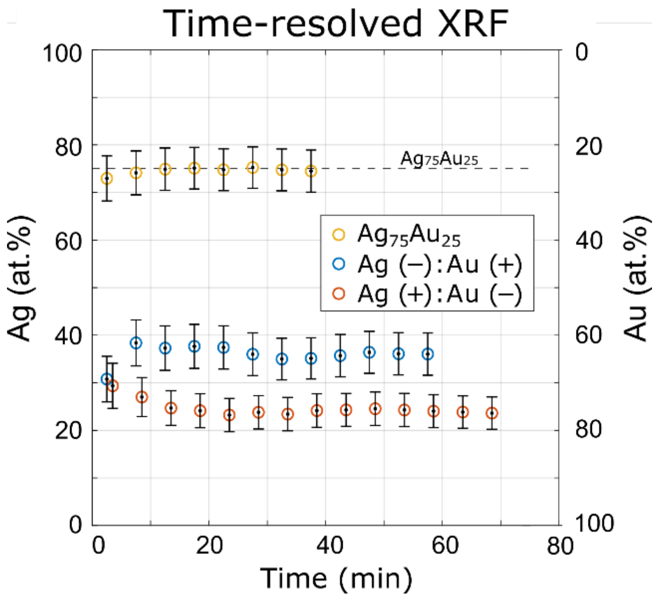


*Figure S8. Time-resolved XRF showing the average particle composition when generated from Ag75Au25 alloys (yellow), and from two pure electrodes, Ag as cathode (−) and Au as anode (+) (blue), and Ag as anode (+) and Au as cathode (−) (red).*

The average composition of NPs produced from alloyed electrodes with the atomic composition Ag75Au25 were measured with 5-minute intervals for 40 minutes and is plotted in Figure S8. Though the initial composition showed a slightly lower Ag composition compared to the specified electrode composition, after 10 minutes of sparking, the nanoparticle composition agreed well with that of the specified. When two pure Ag and Au electrodes were used in combination, the nanoparticle composition varied more over time, especially during the first 10 minutes.

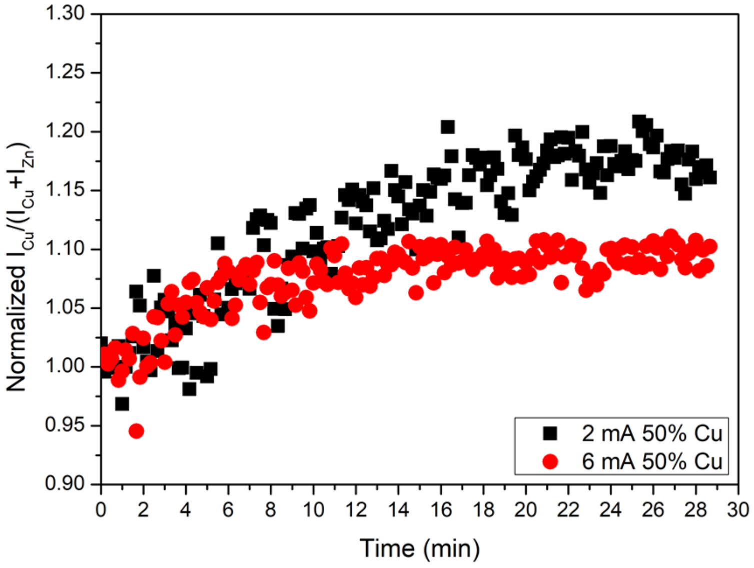


*Figure S9. The normalized optical emission spectroscopy signal from Cu divided by the intensity of both Cu and Zn. The signals are shown over the first 28 minutes of spark ablation in Ar using $Cu_{50}Zn_{50}$ and a charging current of either 2 mA (dark blue squares) or 6 mA (red circles).*

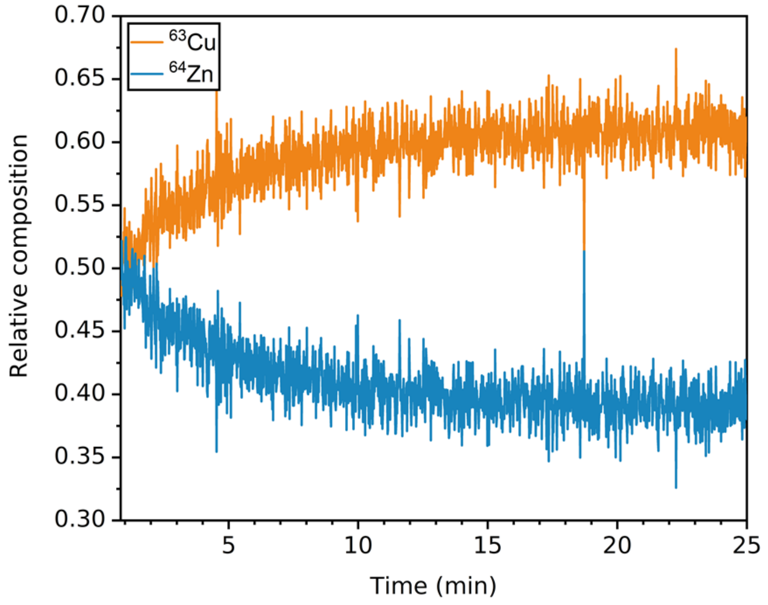


*Figure S10. The Cu and Zn signals from ICP-MS plotted relative to the total signals from Cu and Zn. The signals are shown over the first 25 minutes of spark ablation in Ar using $Cu_{50}Zn_{50}$ and 6 mA as charging current.*

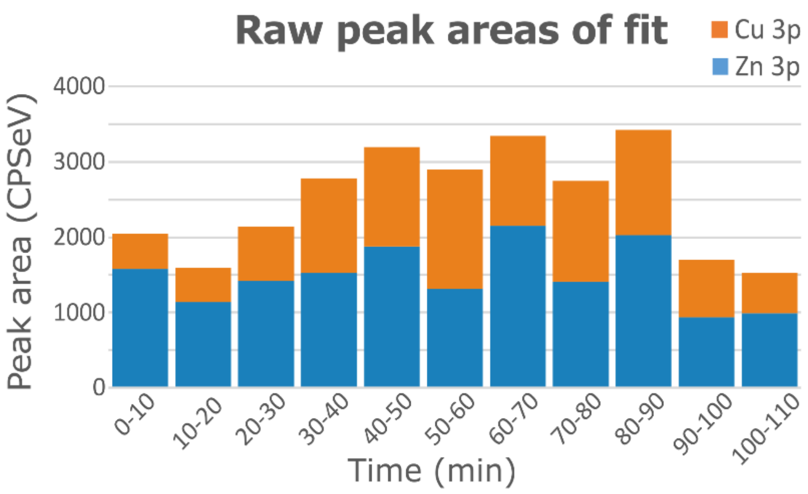


*Figure S11. Peak areas from the XPS fits of the Cu 3p and Zn 3p photoelectron peaks.*

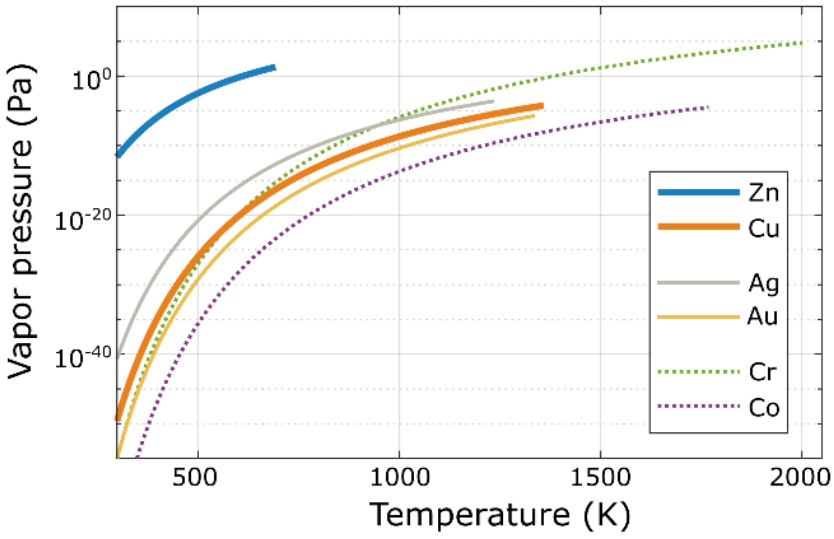


*Figure S12. Vapor pressures of Zn, Cu, Ag, Au, Cr, and Co depending on temperature, and shown up to their individual melting points. Data from* [1]

Figure S12 shows the vapor pressure of a few metals and how it depends on temperature. The Cu–Zn material system exhibits pronounced differences in both melting point ($T_{MCu}$: 1084°C, $T_{MZn}$: 420°C) and vapor pressure ($p_{Zn}/p_{Cu} \approx 10^{32}$ at 350 K) compared to the Ag–Au ($p_{Ag}/p_{Au} \approx 10^{12}$ at 350 K) or the Cr–Co ($p_{Cr}/p_{Co} \approx 10^{10}$ at 350 K) materials systems. Investigations of the Ag–Au and Cr–Co materials systems have not shown similar discrepancies of nanoparticle and electrode composition, though it has not been investigated over time [2, 3], except for the time-resolved XRF of $Ag_{75}Au_{25}$ (Figure S8) which showed very little variation in average nanoparticle composition over time.

## Diffusion in CuZn alloys

It has been shown that in molten brass, containing 10.53 wt.% Zn, Zn atoms can diffuse to the surface and evaporate until, after 2 hours, almost all Zn is gone [4]. The interdiffusion coefficients ($\tilde{D}$) of the Cu–Zn system span approximately $10^{-16}$ m$^2$/s up to $10^{-9}$ m$^2$/s depending on temperature and composition [5]. The diffusion length ($L \sim \sqrt{2\tilde{D}t}$) in molten brass during a spark duration of 5 µs, can range up to ~100 nm, which might favour Zn diffusion and evaporation from a molten pool on the electrode surface. However, the average surface temperature of the electrodes should remain far below (< 100 °C) the melting point of brass (~900-1000 °C) for electrode diameters employed here (3 mm) [6]. Considering that the diffusion length for 1 hour would result in ~850 nm, and that a typical spark erosion crater can be several µm wide and similarly deep [7], this suggests that diffusion from deeper within the electrode cannot be the primary driver for resupplying Zn atoms to the electrode surface for particle formation. Rather, we hypothesise that ablation of partially new electrode material beneath the Cu-rich surface is the primary driver.

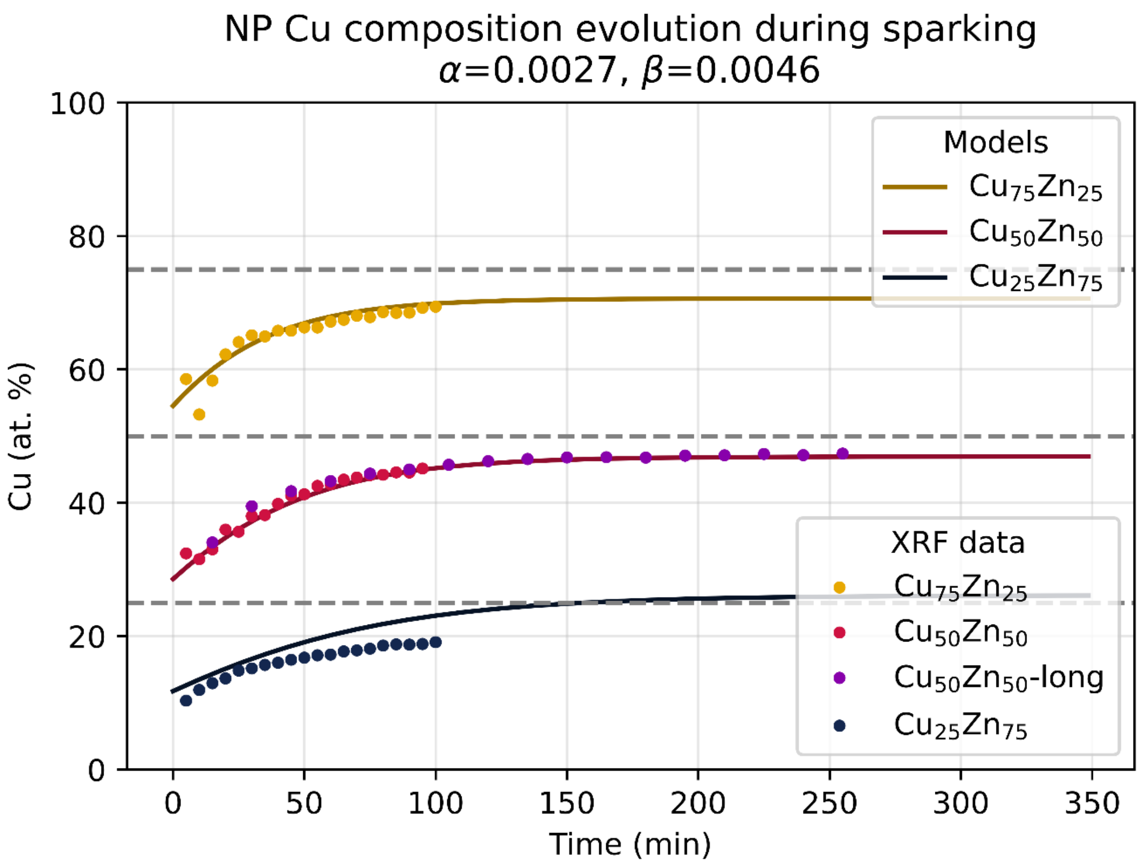


*Figure S13. Temporal evolution of particle composition in terms of Cu content. Simulated models/fits have solid lines, XRF data are filled circles. Dashed lines indicate the original electrode compositions. A steady state composition is reached, though it does not always coincide exactly with that of the original electrode composition. Beta is set larger than alpha.*

The main assumptions of the semi-quantitative ablation model are:

1) Each spark ablates a constant amount of material (described by a parameter α) but only a fraction of it will be released to the gas phase and result in nanoparticles (~0.5 %) [8]. The "release parameters" are different for Zn and Cu ($r_{Zn}$ and $r_{Cu}$, respectively). Their ratio is set to 2.5 ($r_{Zn}$ = 2.5 $r_{Cu}$)
2) The amount of ablated material that does not form NPs can be redeposited on the electrode surface and hence change its local composition. In the model, this is described formally by the terms 1-$r_{Zn}$ and 1-$r_{Cu}$. It is important to note that uneven enrichment is not accounted for; the redeposited material is assumed to be homogeneously distributed within the ablation spot.
3) In case of preferential ablation of Zn, Zn would be fully depleted after a certain number of sparks, so one needs to account for some contribution from the bulk as well, which is assumed to be of constant composition. This is formally done by "mixing" the surface layer with the bulk, described by a parameter β (matched with the long $Cu_{50}Zn_{50}$ data, resulting in approximately two times α).

Below are the main equations in the model, with *n* as the number of sparks:

$x_{Cu}$ is the electrode surface Cu content, which is first set by the ablation stage:

$$x_{Cu\_ablation} = \frac{x_{Cu_{n-1}}(1 - \alpha r_{Cu})}{x_{Cu_{n-1}}(1 - \alpha r_{Cu}) + (1 - x_{Cu_{n-1}})(1 - \alpha r_{Zn})}$$

then by mixing with the bulk:

$$x_{Cu_n} = (1 - \beta)x_{Cu\_ablation} + \beta x_{Cu\_bulk}$$

The gas phase composition, i.e., the nanoparticle composition (in terms of Cu content in at. %):

$$g_{Cu_n} = \frac{\alpha r_{Cu} x_{Cu_n}}{\alpha r_{Cu} x_{Cu_n} + \alpha r_{Zn}(1 - x_{Cu_n})}$$

By setting $r_{Cu}$ to unity, the model can be fitted using two main parameters: α and β. Here, α mainly determines the steepness of the initial composition change, since it controls how much material is affected by ablation during each spark. In contrast, β mainly controls the long-time or steady-state composition, since it describes the extent to which the depleted surface layer is replenished or mixed with material of bulk composition.

If β is large, the surface composition is more efficiently driven back toward the original bulk alloy composition. This leads to a final particle composition that contains relatively more Zn. If β is small, the Zn-depleted surface is less efficiently replenished, and the steady-state particle composition becomes more Cu-rich.

Initially, α was set equal to β for simplicity and to make the model more robust by reducing the number of free parameters. However, this constraint did not provide satisfactory agreement with the long-time XRF data. Therefore, α and β were separated. This allows the model to distinguish between the rate of preferential ablation and the rate of effective surface renewal.

When α and β are fitted to the long-time $Cu_{50}Zn_{50}$ measurements, the model also gives reasonably good agreement with the $Cu_{25}Zn_{75}$ and $Cu_{75}Zn_{25}$ data. This suggests that the model captures the general mechanism: preferential Zn removal leads to Cu enrichment of the surface, while replenishment from the underlying alloy prevents complete Zn depletion and gives rise to a dynamic steady state. It should be noted that the dashed lines in Figure S13 indicate the nominal electrode compositions, not theoretical limits for the nanoparticle composition. Since preferential Zn ablation enriches the electrode surface in Cu, the nanoparticle composition can, in principle, become more Cu-rich than the nominal feedstock composition. The slight overestimation of the steady-state Cu content for the $Cu_{25}Zn_{75}$ case therefore does not represent an unphysical result. Rather, it reflects the simplification of using one common parameter set for all alloy compositions. In reality, the effective preferential ablation is likely composition dependent due to differences in alloy phase, melting behavior, vapor pressure, and other thermophysical properties.

In the fitted case, β is larger than α. Within the framework of the model, this means that the electrode surface is replenished toward the bulk composition more efficiently than would be expected from the ablation fraction alone. Physically, this may indicate that each spark modifies a larger near-surface volume than the fraction of material that is ultimately released as gas-phase nanoparticles. Local melting, redeposition of material, exposure of fresh alloy, and short-range near-surface mixing may therefore replenish the Zn-depleted surface more efficiently than assumed in the simplest case where α=β.

However, the model should still be regarded as semi-quantitative. Although it reproduces the overall trends, it does not fit all alloy compositions perfectly. This limitation likely arises because the same

effective parameter set is applied to all alloy compositions, while several physical processes—including preferential ablation, surface renewal, and redeposition—are likely composition dependent. In reality, these processes may vary with alloy composition, melting point, vapor pressure, hardness, local spark morphology, and redeposition efficiency. Therefore, β should not be interpreted as a direct physical diffusion or mixing coefficient, but rather as an effective parameter describing the combined influence of these processes.

# References


1. C. B. Alcock, V. P. Itkin and M. K. Horrigan, Canadian Metallurgical Quarterly, 1984, 23, 309-313.
2. L. Jönsson, M. Snellman, A. Eriksson, M. Kåredal, L. R. Wallenberg, S. Blomberg, A. Kohut, L. Hartman and M. Messing, Journal of Aerosol Science, 2024, 177.
3. N. S. Tabrizi, Q. Xu, N. M. van der Pers, U. Lafont and A. Schmidt-Ott, Journal of Nanoparticle Research, 2009b, 11, 1209-1218.
4. M. Wilk, T. Matula, L. Blacha, A. Smalcerz and J. Labaj, Materials, 2023, 16.
5. C. M. Eastman, Q. Zhang and J.-C. Zhao, Journal of Phase Equilibria and Diffusion, 2020, 41, 642-653.
6. C. Loizidis, K. C. Petallidou, A. Maisser, S. Bezantakos, T. V. Pfeiffer, A. Schmidt-Ott and G. Biskos, Aerosol Science and Technology, 2024, 58, 1421-1431.
7. A. Kohut, M. Wagner, M. Seipenbusch, Z. Geretovszky and G. Galbács, Journal of Aerosol Science, 2018, 119, 51-61.
8. J. Feng, L. Huang, L. Ludvigsson, M. E. Messing, A. Maisser, G. Biskos and A. Schmidt-Ott, The Journal of Physical Chemistry C, 2016, 120, 621-630.
